# A dual-function liquid electrolyte additive for high-energy non-aqueous lithium metal batteries

**DOI:** 10.1038/s41467-022-28959-5

**Published:** 2022-03-11

**Authors:** Yuji Zhang, Yuan Wu, Huiyi Li, Jinghao Chen, Danni Lei, Chengxin Wang

**Affiliations:** State Key Laboratory of Optoelectronic Materials and Technologies, School of Materials Science and Engineering, Sun Yat-sen (Zhongshan) University, Guangzhou, 510275 China

**Keywords:** Batteries, Batteries, Characterization and analytical techniques, Energy, Electrochemistry

## Abstract

Engineering the formulation of non-aqueous liquid electrolytes is a viable strategy to produce high-energy lithium metal batteries. However, when the lithium metal anode is combined with a Ni-rich layered cathode, the (electro)chemical stability of both electrodes could be compromised. To circumvent this issue, we report a combination of aluminum ethoxide (0.4 wt.%) and fluoroethylene carbonate (5 vol.%) as additives in a conventional LiPF_6_-containing carbonate-based electrolyte solution. This electrolyte formulation enables the formation of mechanically robust and ionically conductive interphases on both electrodes’ surfaces. In particular, the alumina formed at the interphases prevents the formation of dendritic structures on the lithium metal anode and mitigate the stress-induced cracking and phase transformation in the Ni-rich layered cathode. By coupling a thin (i.e., about 40 μm) lithium metal anode with a high-loading (i.e., 21.5 mg cm^−2^) LiNi_0.8_Co_0.1_Mn_0.1_O_2_-based cathode in coin cell configuration and lean electrolyte conditions, the engineered electrolyte allows a specific discharge capacity retention of 80.3% after 130 cycles at 60 mA g^−1^ and 30 °C which results in calculated specific cell energy of about 350 Wh kg^−1^.

## Abstract (web summary)

Lithium metal batteries suffer from poor (electro)chemical stability of the electrodes during prolonged cycling. Here, the authors report a dual function liquid electrolyte additive to form protective interphases on both electrodes to produce lab-scale high energy lithium metal batteries.

## Introduction

Lithium-ion batteries (LIBs) with lithium-intercalated graphite anodes have been widely used since their commercialization in the 1990s^1,2^. However, with their lower specific capacity (372 mAh g^−^^1^), graphite anodes can hardly meet the practical needs of highly demanding modern devices such as electric vehicles^3,4^. The lithium metal anode (LMA) is considered the most promising since it has a theoretical specific capacity of 3862 mAh g^−^^1^ and the lowest reduction potential (−3.040 V versus the standard hydrogen electrode (SHE))^5–7^. Replacing graphite anodes in LIBs with LMA and combining them with high-capacity cathodes, such as Ni-rich layered cathode would significantly boost their energy density. High specific content lithium metal batteries (LMBs) of over 300 Wh kg^−^^1^ require an integrated Li anode with a thickness of less than 50 μm, a limited amount of electrolyte (electrolyte weight to cathode capacity ratio (E/C) < 4) and a high areal-capacity cathode (> 4 mAh cm^−^^2^)^8,9^. However, in practise, the performance of LMBs is often unsatisfactory due to the high reactivity of conventional carbonate-based electrolytes with both Ni-rich cathodes LiNi_0.8_Co_0.1_Mn_0.1_O_2_ (NCM) and LMA^10,11^. Side reactions between LMA and carbonate-based electrolytes result in an unsatisfactory Coulombic efficiency (CE) and the growth of unfavourable Li deposition morphologies (e.g., dendrites), which significantly decrease battery performance and cause potential safety issues^12^. In addition, Ni-rich NCM cathodes are impacted by phase transformation, stress-corrosion cracking and transition metal dissolution during long cycling^13^. Therefore, the design of improved nonaqueous liquid electrolyte solutions to construct a consistent and robust solid electrolyte interphase (SEI) at the anode and a cathode-electrolyte interphase (CEI) at the cathode is critical to the practical utilization of high specific energy LMBs^14,15^.

Recently, significant efforts have been made to optimize electrolytes for better compatibility with both Ni-rich NCM cathodes and LMAs^16–18^. Highly or locally concentrated electrolytes facilitate the formation of a salt-derived SEI or CEI, potentially improving the cycling stability of Li | |NCM batteries^19–21^. However, certain electrolyte challenges remain to be addressed before commercialization, such as their high viscosity, insufficient separator wettability, low ionic conductivity and high cost. All-fluorinated electrolytes^22^ and fluorinated ether-based electrolytes^23^, such as those containing LiF, have been demonstrated to form compounds with a preferable SEI or CEI, but evaluation under practical conditions (specific energy > 300 Wh kg^−1^) is warranted. Sulfonamide-based electrolytes^24^ and phosphate-based electrolytes^25^ can stabilize the cycling of high-voltage LMBs, but the use of high-cost lithium bis(fluorosulfonyl)imide (LiFSI) to lower the reactivity of electrolytes with LMAs cannot be avoided^26,27^. The dual-additive strategy (utilizes the synergistic effect of two different additives) has been proven to improve the performance of Li | |NCM cells by regulating the CEI and SEI structures^8,11^, but Li-containing additives are also expensive^28^. Outside of electrolyte engineering, the application of an Al_2_O_3_ surface coating on a high-voltage cathode and Li metal anode is a common approach to improve the stability between active materials and electrolytes. On the cathode, Al_2_O_3_ coatings can typically scavenge HF, limit transition metal dissolution and regulate the composition of the CEI^29^. On the anode, Al_2_O_3_ thin films prevent side reactions with electrolytes and induce homogeneous Li nucleation^30^. Commonly, atomic layer deposition^31,32^, radiofrequency magnetron sputtering^33^ and wet chemistry^34,35^ are used to coat an Al_2_O_3_ protective film on electrodes. However, these methods require a high temperature; additionally, the process may be rather complex and expensive. Moreover, some of these methods cannot guarantee the uniformity of the coating^36^. Therefore, a low cost and effective method is needed to realize practical LMBs with a high specific energy.

Herein, we design a nonaqueous electrolyte solution by adding 0.4 wt.% aluminum ethoxide (Al(EtO)_3_) nanowires into a conventional LiPF_6_-containing carbonate-based electrolyte solution. Al(EtO)_3_ is prepared by a selective dealloying method^37^. With the synergistic effect of Al(EtO)_3_ and fluoroethylene carbonate (FEC), Al_2_O_3_-containing interphases that provide robust and high ionic conductivity protection are formed, which is beneficial to both Li metal anodes and Ni-rich NCM cathodes. Indeed, when the Al(EtO)_3_-containing electrolyte solution is tested in Li | |LiNi_0.6_Co_0.2_Mn_0.2_O_2_ (NCM622) coin cell configuration, improved cycling stability and rate performance can be noticed. Also, by pairing the thin Li anode (~40 μm) with the commercial cathode LiNi_0.8_Co_0.1_Mn_0.1_O_2_ (NCM811) (21.5 mg cm^−2^), we demonstrate a specific energy of approximately 350 Wh kg^−1^ at the full cell level.

## Results

### Physicochemical characterization of the electrolyte solutions

**Fig. 1:**
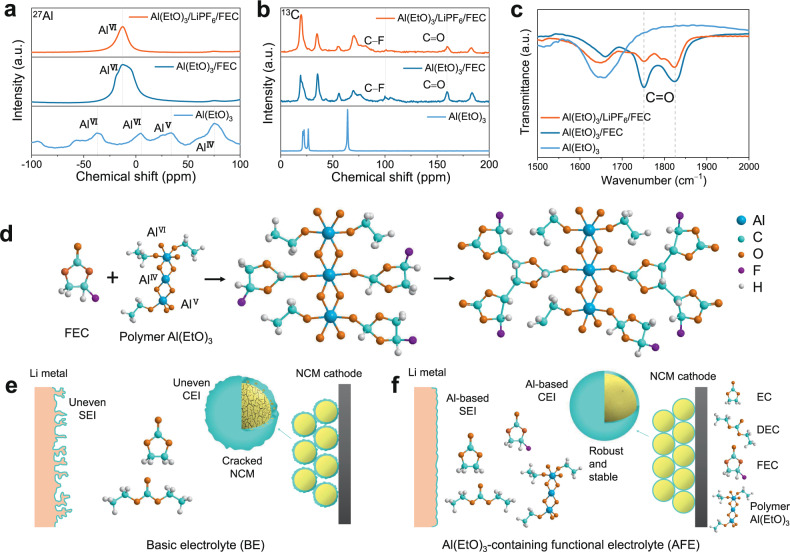
Physicochemical characterizations of the electrolyte solutions and additives. ^27^Al (**a**) and ^13^C (**b**) Solid-state NMR and **c** Solid FTIR spectra of pure Al(EtO)_3_ and the precipitates obtained from two different solutions. **d** Proposed Al(EtO)_3_/FEC reaction mechanism. Schematic illustration of the protection mechanism of the SEI and CEI formed in BE (**e**) and AFE (**f**).

The color of the Al(EtO)_3_-containing electrolyte (AFE, 0.4 wt.% Al(EtO)_3_ and 5 vol.% FEC in 1 M LiPF_6_/ethylene carbonate (EC) and diethyl carbonate (DEC) (1:1 by volume)) in a polyolefin bottle that was placed in an Ar-filled glove box for five days at 30 °C and 60 °C turns from transparent to brown and produces an acidic gas (Supplementary Figs. 1 and 2), suggesting that side reactions were occurring. All the cells were tested with freshly prepared electrolyte. To explore the reaction mechanism of Al(EtO)_3_ and FEC, we prepared Al(EtO)_3_/LiPF_6_/FEC (3.2 wt.% Al(EtO)_3_ + 1.0 M LiPF_6_ in FEC) and Al(EtO)_3_/FEC (3.2 wt.% Al(EtO)_3_ in FEC) solutions in a polyolefin bottle and placed them in an Ar-filled glove box for five days at 60 °C. Then, we took the precipitate and supernatant for further characterization. The ^27^Al magic angle spinning solid-state nuclear magnetic resonance (NMR) spectra of Al(EtO)_3_ show several resonance peaks centered at approximately 75, 33, 4 and −36 ppm. Among them, the peaks at 75 and 33 ppm correspond to 4- (Al^IV^) and 5-coordinated aluminum (Al^V^), respectively, while the peaks at 4 and -36 ppm correspond to 6-coordinated aluminum (Al^VI^)^38,39^. These results indicate the formation of Al(EtO)_3_ polymer networks. The ^27^Al MAS solid-state NMR spectra of the Al(EtO)_3_/LiPF_6_/FEC and Al(EtO)_3_/FEC precipitates show a resonance peak centered at –12 ppm, which may correspond to 6-coordinated aluminum (Al^VI^) (Fig. 1a). Compared with the Al(EtO)_3_/FEC precipitate, the Al(EtO)_3_/LiPF_6_/FEC precipitate shows weaker peaks for C–F bonds (100 ppm) and C=O (159 ppm)^40^ in the ^13^C spectrum, indicating that the LiPF_6_ may catalyse the reaction of Al(EtO)_3_ and FEC (Fig. 1b). As shown in the solid Fourier transform infrared (FTIR) spectra, the C=O peaks (1751 cm^−^^1^ and 1825 cm^−^^1^) of the precipitate of the Al(EtO)_3_/LiPF_6_/FEC solution are weaker than those of the Al(EtO)_3_/FEC solution (Fig. 1c). This further confirms the catalytic properties of LiPF_6_. Although the color of the Al(EtO)_3_/LiPF_6_/FEC solution changes significantly in the supernatant after five days of storage, the C−F and C=O signals show no marked changes in the NMR and FTIR spectra (Supplementary Fig. 3). This may be because most of the reaction products settle at the bottom and their concentrations are low in the supernatant. The possible reaction mechanism of Al(EtO)_3_ and FEC is proposed in Fig. 1d. The C=O bond of FEC is broken, and oxygen atoms are directly connected to the Al atom of Al(EtO)_3_, followed by polymerization of FEC and the loss of HF. With the lowest heat absorption and the highest decomposition temperature, the reaction products of Al(EtO)_3_ and FEC catalyzed by LiPF_6_ have good thermal stability (Supplementary Figure 4). We speculate that the reaction products with a three-dimensional structure and high thermal stability would contribute to the formation of uniform and robust solid-electrolyte interphases at the anode and cathode. Based on the findings discussed above, we schematically illustrate in Figs. 1e, 1f the possible SEI and CEI morphologies obtained employing the conventional electrolyte and Al(EtO)_3_-containing functional electrolyte (AFE) in Li | |NCM cells.

### Morphological and mechanical characterizations of lithium depositions on Cu substrates

**Fig. 2:**
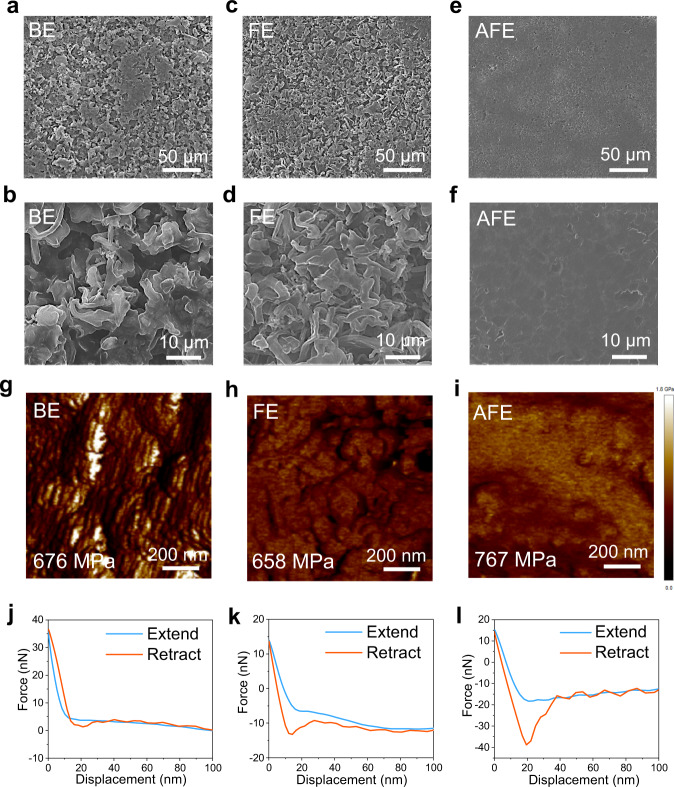
Micromorphology and SEI properties of Li metal deposited onto a Cu substrate. SEM images (**a**–**f**), Young’s modulus maps (**g**–**i**) and force-displacement curves (**j**-**l**) of the deposited lithium metal using the BE (**a**, **b**, **g** and **j**), FE (**c**, **d**, **h** and **k**) and AFE (**e**, **f**, **i** and **l**) at current densities of 0.5 mA cm^−^^2^ and 3 mAh cm^−^^2^. “Extend” and “retract” means the direction of motion of the cantilever with respect to the sample.

To better understand the Li deposition behavior in different electrolytes, we assembled Li | |Cu coin cells and used scanning electron microscopy (SEM) for observation. All Li | |Cu coin cells were operated at a fixed current density of 0.5 mA cm^−2^ with a specific areal capacity limitation of 3 mAh cm^−2^ before cell disassembly and postmortem SEM electrode measurements. For the cells with a basic electrolyte (BE, 1 M LiPF_6_ in EC/DEC (1:1 by volume)), the top views of the electroplated Li metal on the surface of the Cu electrode exhibit a typical dendritic morphology (Fig. 2a and b). The dendritic Li metal increases the reaction area with the electrolyte; thus, the electrolyte is quickly consumed, “dead Li” (i.e., lithium particles that are electronically disconnected from the current collector) is formed, and the cell exhibits a short cycle life. Uneven and loose Li dendrites are observed even when 5% FEC is added to the BE (FEC-containing electrolyte (FE)) (Fig. 2c and d). In sharp contrast, uniform and dense Li deposits without dendrite are obtained with the use of AFE (Fig. 2e and f).

During lithiation/delithiation, SEI deformation is inevitable. Therefore, toughness and adhesion are essential to ensure the integrity of the SEI film and inhibit Li dendrite formation^41,42^. Through atomic force microscopy (AFM) evaluation, the Young’s modulus of the SEI formed in the AFE is 767 MPa, which is higher than that in the BE (676 MPa) and FE (658 MPa) (Fig. 2g-i), indicating a tougher SEI film that effectively inhibits the formation of Li dendrites. In the force-displacement curves, the SEI interphase formed in the AFE has the highest adhesion force among the three groups, which effectively enhances the interfacial stability and promotes the rapid transport and uniform deposition of Li^+^ (Fig. 2j-l).

### Physicochemical characterization of the lithium metal anode

**Fig. 3:**
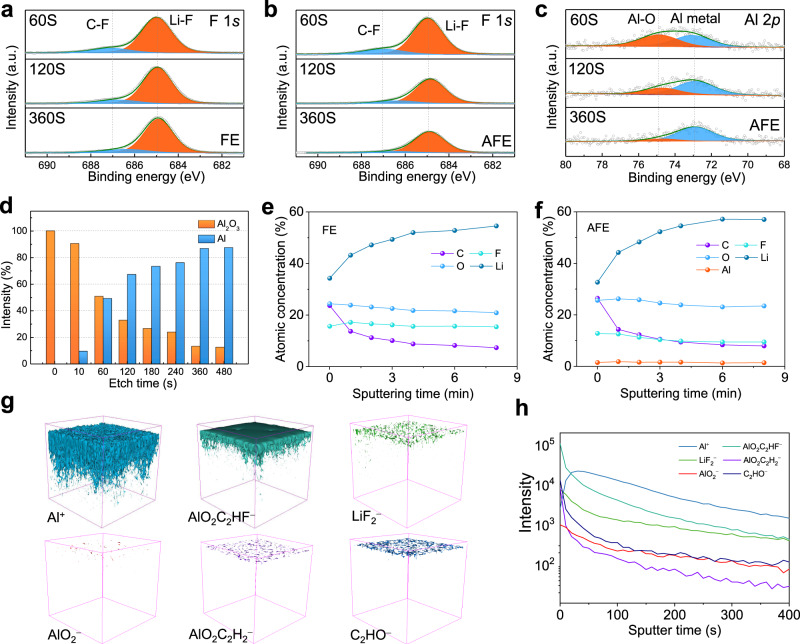
Ex situ postmortem physicochemical characterizations of lithium metal electrodes cycled in symmetrical Li | |Li cells. XPS F 1 *s* spectra (**a** and **b**), XPS Al 2 *p* spectra (**c**), Al-O/Al ratio as the XPS sputtering time is increased in the AFE (**d**), atomic ratio of elements in the SEI at different XPS sputtering times (**e** and **f**). TOF-SIMS 3D reconstruction of the sputtered volume (**g**) and the depth profiling of several secondary ion fragments (**h**) of Al^+^, AlO_2_C_2_HF^−^, LiF_2_^−^, AlO_2_^−^, AlO_2_C_2_H_2_^−^ and C_2_HO^−^ on the Li surface. Li | |Li cycling was obtained at a current density of 1 mA cm^−^^2^ and an areal capacity of 1 mAh cm^−^^2^ for each plating or stripping step.

The synergistic effect of dual additives (Al(EtO)_3_/FEC) on the chemical composition of the SEI film on a cycled Li anode (at a current density of 1 mA cm^−2^ and an areal capacity of 1 mAh cm^−2^ for 10 cycles; the voltage of the cell was 0 V before disassembly) was studied by using in-depth X-ray photoelectron spectroscopy (XPS) (Fig. 3a-f). The high-resolution F 1 *s* XPS spectra of the SEI film using FE and AFE had peaks at 684.9 eV and 687.0 eV^43^, respectively, and these peaks are assigned to LiF and C−F species as the main reduction products of FEC (Fig. 3a and b). With increasing Ar^+^ etching time, the content of LiF drops in the AFE, whereas it remains unchanged in the FE. The proportion of F in the AFE is lower than that in the FE. This confirms that the reaction of Al(EtO)_3_ and FEC releases HF. The high-resolution Al 2 *p* XPS spectra of the SEI in the AFE show the presence of Al (72.9 eV)^44^, which confirms that the reaction product of Al(EtO)_3_ and FEC can be reduced to Al metal. In addition, Al-O species are detected based on the peak at 74.9 eV (Fig. 3c, Supplementary Fig. 5). To verify the presence of Al_2_O_3_ and exclude the influence of Ar^+^ on Al_2_O_3_, we conducted in-depth XPS on pure Al_2_O_3_ powder under the same conditions. With increasing Ar^+^ etching time, the peak at 74.9 eV corresponding to Al_2_O_3_^45^ slightly shifts to a higher binding energy, which confirms that Al_2_O_3_ is not be reduced to Al metal by Ar^+^ (Supplementary Fig. 6). With increasing Ar^+^ etching time, the amount of Al-O species decreases while that of Al metal increases, which means that the inner SEI layer contains more Al metal (Fig. 3d). The Al/Al_2_O_3_ composite structure can effectively reduce the consumption of Li^+^ during SEI film formation^46^. The total atomic content of Al remains at 1.5% of all elements and does not change with the etching depth as shown in Fig. 3e, f; this result indicates the uniform distribution of aluminum in the SEI film. A Li metal signal (52.4 eV)^47^ is detected in the AFE, as shown in the Li 1 *s* spectra (Supplementary Fig. 7a and b), indicating the high density of the SEI and the intimate contact between the SEI and Li metal anode. The C 1 *s* spectra in the two electrolytes are basically the same (Supplementary Fig. 7c and d). Time-of-flight secondary ion mass spectrometry (TOF-SIMS) measurements were performed to provide more information about the composition of the SEI (Fig. 3g and h, Supplementary Figs. 8 and 9). The interphase products, including the aluminum-containing species (represented by Al^+^, AlO_2_C_2_HF^−^, AlO_2_^−^ and AlO_2_C_2_H_2_^−^), inorganic species (represented by LiF_2_^−^ and CO_3_^−^) and organic species (represented by C_2_HO^−^ and CHO_2_^−^), appear at the SEI on the Li surface. AlO_2_C_2_HF^−^ and AlO_2_C_2_H_2_^−^ are representative of the fragments of the reaction products shown in Fig. 1d, which further indicates that the speculated reaction mechanism of Al(EtO)_3_ and FEC (Fig. 1d) is reasonable. Compared to conventional SEI components (such as Li_2_CO_3_, Li_2_O and LiOH)^48^, the spatial distribution of aluminum-containing species is deeper, indicating that aluminum-containing species are closer to the LMA, which is consistent with the XPS results. Contact with the LMA is conducive to the protective effect of the aluminum-based solid electrolyte interphase on the electrode. In other words, the synergistic effect of Al(EtO)_3_ and the FEC provides a robust SEI containing Al metal and Al−O species that contributes to the high stability of the LMA during long-term cycling.

### Electrochemical measurements and analyses of Li||Cu and Li||Li coin cells

**Fig. 4:**
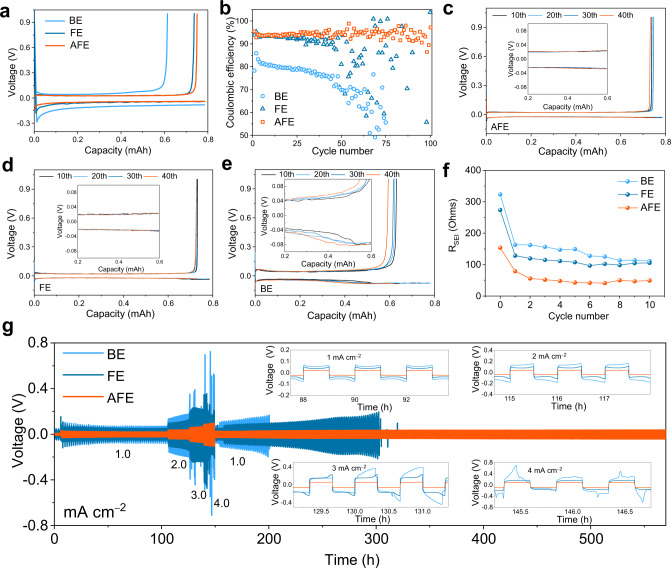
Electrochemical characterizations of the Li | |Cu and Li | |Li cells with different electrolytes. Initial charge–discharge curves (**a**), Coulombic efficiency (**b**) and voltage profiles (**c**, **d**, and **e**) of the Li | |Cu cells. **f** R_SEI_ of the Li | |Li cells with an increasing cycle number. **g** Rate capability of the Li | |Li cells with different electrolytes. Inset is the enlarged figure of the curves.

To further verify that the SEI can improve the reversibility of Li deposition, we assembled Li | |Cu cells with the BE, FE and AFE. All cells were discharged into a fixed capacity of 1 mAh cm^−2^ and then charged to 1 V to strip the Li off at a current density of 0.5 mA cm^−2^. As shown in Fig. 4a, the cell with the AFE has the lowest Li deposition potential due to the lithiophilic property of the Al metal in the SEI. Although metallic Li should easily react with elemental Al to form Li-Al alloys, no plateaus of Li-Al alloying/dealloying are observed in the stripping-plating voltage profiles of the Li | |Cu cells with the AFE^49^, which is similar to that with the BE (Supplementary Fig. 10). Thus, we speculate that the amount of metallic Al contained in the SEI is insufficient to trigger an alloy reaction with the lithium ions coming from the electrolyte. The initial CE of the cell with the BE is 78.3% and quickly drops to less than 50.0% after 70 cycles (Fig. 4b). In the FE, the CE improves during the initial 40 cycles and then widely fluctuates. In contrast, the CE of the cell with the AFE remains stable for over 100 cycles with an average of 94.0%. The corresponding voltage profiles shown in Fig. 4c-e indicate that the cell with the AFE has stable polarization without a sharp increase during the Li plating/stripping process, which is improved if compared with the cells assembled using the BE and FE.

To better understand the stability of the SEI, we measured and simulated the electrochemical impedance spectroscopy (EIS) results of Li | |Li symmetrical cells after different cycle numbers at the current density of 1.0 mA cm^−2^ and capacity of 1 mAh cm^−2^. The semicircle of the Nyquist plots in the high-frequency region represents Li^+^ migration through the SEI^50^. As reported in the literature, Li metal is highly reactive, both chemically and electrochemically, with organic-based liquid electrolytes^51^. The R_SEI_ of the uncycled cell in the BE is as high as 323.4 Ohms, which suggests that the initial SEI is uneven. The R_SEI_ is slightly lower in the FE (274.2 Ohms), and markedly lower in the AFE (154.0 Ohms) owing to the reaction between Al(EtO)_3_ and FEC in the absence of an electric field (Fig. 4f). After two cycles, the R_SEI_ in the AFE drops to one-third of its original value (49.0 Ohms) and remains stable during subsequent cycles. In contrast, the R_SEI_ values in both the BE and FE heavily fluctuate, and the average values are twice that of the AFE (Supplementary Fig. 11). This reinforces our conclusion that the product of the FEC/Al(EtO)_3_ reaction contributes to uniform and robust SEI formation and thus more efficient and uniform Li-ion transport.

We analyzed and compared the cycling stability and high-rate capability of Li | |Li symmetrical cells in three electrolytes (Fig. 4g). The Li | |Li cells cycled in the BE and FE quickly fail when the current density is increased from 1 to 3 and 4 mA cm^−2^, respectively. In contrast, the Li | |Li cell with the AFE cycles steadily for 550 h and exhibits a stable voltage profiles with low electrode polarization even when the current density is increased to 4.0 mA cm^−^^2^. This further endorses the fact that the robust SEI that forms in the AFE improves the interfacial compatibility, thus enabling highly reversible Li deposition/dissolution.

### Electrochemical characterization of the Li||NCM coin cells

**Fig. 5:**
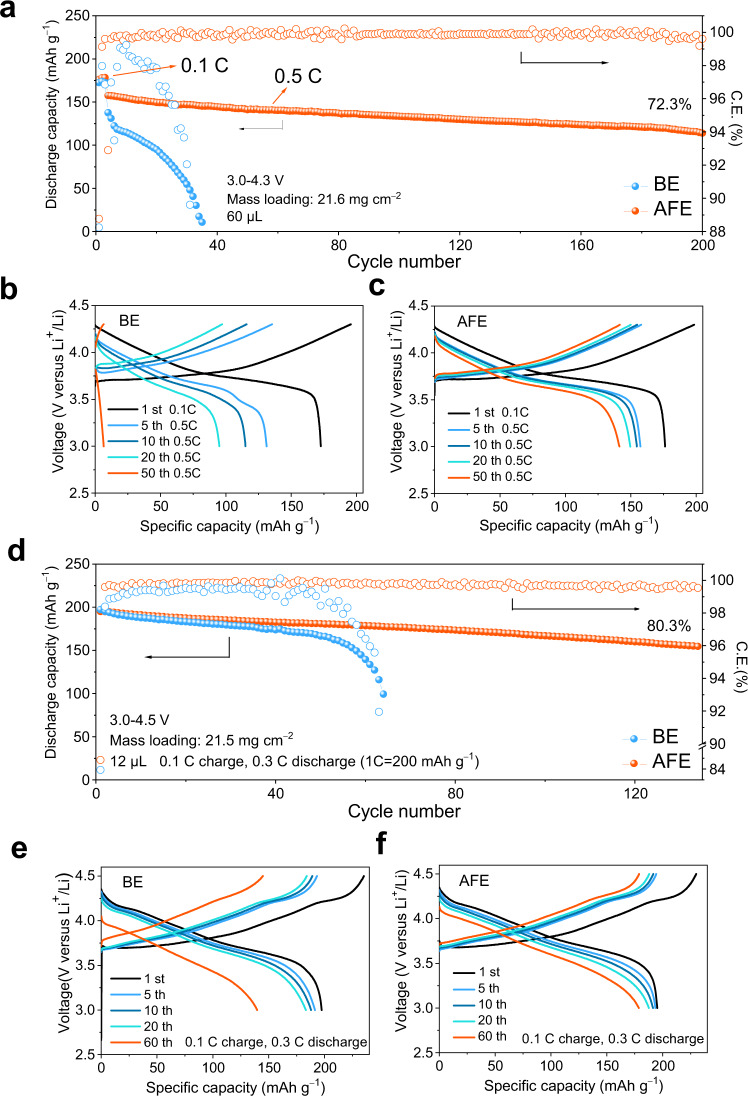
Electrochemical energy storage of the Li | |NCM cells with different electrolytes. Cycling stability (**a**) and corresponding voltage profiles of the Li | |NCM622 cells using the BE (**b**) and AFE (**c**) at a 4.3 V cut-off voltage. The discharging/charging current were 19 mA g^−^^1^/19 mA g^−^^1^ (0.1 C/0.1 C) for the first three cycles and 95 mA g^−^^1^/95 mA g^−^^1^ (0.5 C/0.5 C) afterwards. Cycling stability (**d**) and corresponding voltage profiles of the Li | |NCM811 cells using the BE (**e**) and AFE (**f**) at a 4.5 V cut-off voltage, and the discharging/charging current were 60 mA g^−^^1^/20 mA g^−^^1^ (0.3 C/0.1 C).

To evaluate the high-voltage cycling performance of AFE, we assembled Li | |NCM622 and Li | |NCM811 full cells. Li | |NCM622 coin cell with a cathode mass loading of 2.4 mg cm^−^^2^ was cycled at 19 mA g^−^^1^, 38 mA g^−^^1^, 95 mA g^−^^1^, 190 mA g^−^^1^, 380 mA g^−^^1^ and 950 mA g^−^^1^ for 5 cycles and finally stabilized at 190 mA g^−^^1^. The cells with the AFE could be cycled up to 1000 cycles delivering a specific discharge capacity of 98.2 mAh g^−^^1^ while the cell with the BE could be cycled only for more than 400 cycles before failure (Supplementary Fig. 12). When the AFE was tested with a commercial cathode with a high mass loading of 21.6 mg cm^−^^2^, the Li | |NCM622 coin cell show a 72.3% capacity retention after 200 cycles at 95 mA g^−^^1^ (Fig. 5a). The voltage profiles of different cycle numbers show that the polarization in the AFE is much smaller than that in the BE (Fig. 5b and c). The charge transfer resistance (R_ct_) and cathode electrolyte interphase resistance (R_CEI_) of the fresh Li | |NCM622 cell with the AFE are almost the same as that with the BE. But after the 60th cycle, both the R_CEI_ (33.9 Ohms) and R_ct_ (36.1 Ohms) of the Li | |NCM622 cell with the AFE (Supplementary Fig. 13a) dramatically decrease while those with the BE (R_CEI_ = 101.8 Ohms, R_ct_ = 91.8 Ohms) significantly increase (Supplementary Figure 13b and Table 1). This indicates that the AFE electrolyte may improve the stability of the Li | |NCM cells. To investigate the effect of the environmental temperature on the cycling behaviour of the cells assembled with various electrolytes, we tested several Li | |NCM622 cells in the 30–60 °C range (Supplementary Fig. 14). The Li | |NCM622 full cell with the BE presents fast capacity attenuation and fluctuations in coulombic efficiency at 40 °C. In contrast, the Li | |NCM622 full cell with the FE can cycle steadily at 50 °C but shows a rapid decrease in capacity and coulombic efficiency at 60 °C. This behaviour indicates that the addition of FEC can improve the electrochemical energy storage performances of the coin cells at temperatures > 30 °C. In addition, the cycling performance of the Li | |NCM622 full cell with the AFE is much better than that with the BE and FE, which confirms that the addition of Al(EtO)_3_ can further promote the thermal stability of carbonate electrolyte. When using a high-loading cathode, lean electrolyte condition and thin lithium metal anode, it is a great challenge to obtain a satisfactory cycle life. In the AFE, full coin cells with a high-loading NCM811 cathode (~4 mAh cm^−2^), thin Li foil (41 μm, N/P ratio of ~2.13) (Supplementary Fig. 15) and limited electrolyte (E/C ratio of ~3.4 g Ah^−1^) can stably cycle 130 times at discharge/charge rates of 60 mA g^−^^1^/20 mA g^−1^ and 4.5 V, while those with the BE rapidly degrade within 60 cycles (Fig. 5d). In addition, the Li | |NCM811 cells with the BE exhibit an increase in polarization (Fig. 5e). In comparison, the Li | |NCM811 cells with the AFE exhibit lower polarization and higher capacity retention (Fig. 5f). It is worth mentioning that the charge voltage plateau at 4.2 V is an intrinsic property of NCM811^17^ rather than being due to the decomposition of electrolyte. No oxidation current peaks from the 3 to 5 V are observed in the linear sweep voltammetry (LSV) results of the BE and AFE (Supplementary Fig. 16). The calculated specific energy of the Li|AFE | NCM811 coin cell is 356.23 Wh kg^−1^ without considering the packing material^8^ (Supplementary Table 2). To further illustrate the advantages of our technology in practical applications, we calculated the energy density of an 18650 cell with our own parameters (346.1 Wh kg^−1^) according to a reported method (Supplementary Table 3)^52,53^. The calculated specific energy values are well aligned with other results reported in the literature (Supplementary Table 4).

### Ex-situ postmortem physicochemical characterizations of the NCM-based electrodes

**Fig. 6:**
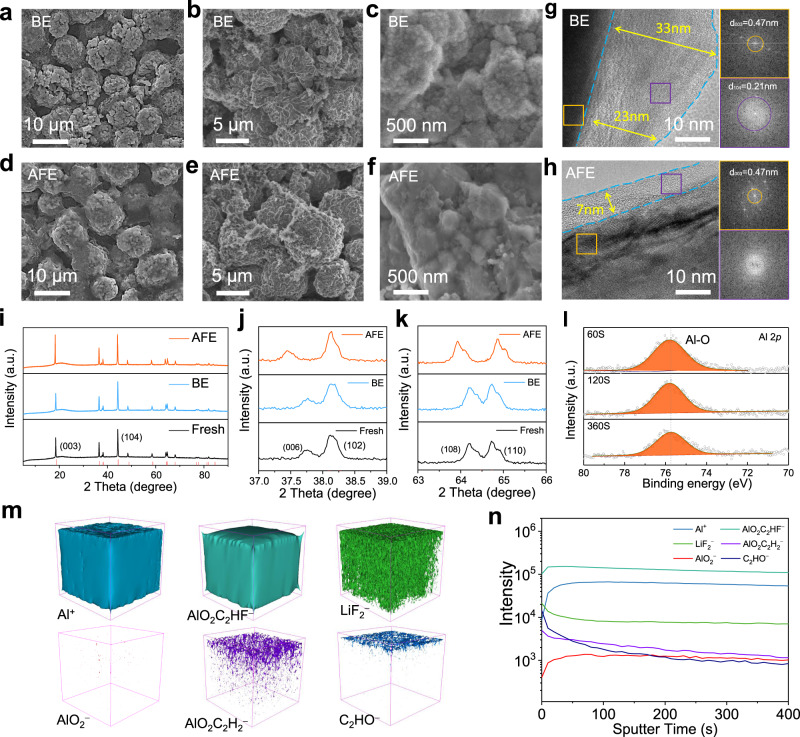
Physicochemical characterization of the cycled high-loading NCM622 cathodes with different electrolytes. SEM images of the NCM622 cathode surface after 100 cycles in the BE (**a**) and AFE (**d**). SEM images of the NCM622 cathodes cross-sectioned after 100 cycles in the BE (**b** and **c**) and AFE (**e** and **f**). TEM analyses of the NCM622 particles after 100 cycles in the BE (**g**) and AFE (**h**). XRD of the NCM622 cathodes (**i**–**k**) before and after 100 cycles in the BE and AFE. XPS spectra of the Al 2 *p* (**l**) spectrum of the NCM622 cathodes in the AFE. TOF-SIMS 3D reconstruction of the sputtered volume (**m**) and the depth profiling of several secondary ion fragments (**n**) of Al^+^, AlO_2_C_2_HF^−^, LiF_2_^−^, AlO_2_^−^, AlO_2_C_2_H_2_^−^ and C_2_HO^−^ on the NCM622 surface after 100 cycles. All the cells were charged and discharged between 3.0 and 4.3 V at 95 mA g^−1^ for 100 cycles, and the voltage of the cell was 3.0 V before disassembly.

To understand the cycling stability mechanisms of the Li | |NCM622 full cells, we further characterized the cycled NCM622 cathodes (charged and discharged between 3.0 and 4.3 V at 95 mA g^−^^1^ for 100 cycles, and the voltage of the cell was 3.0 V before disassembly) with high mass loading in different electrolytes (Fig. 6). Severe cracks are observed in the NCM622 primary particles and many small fragments are observed in the NCM622 secondary particles with the BE (Fig. 6a-c, Supplementary Fig. 17a). The fractured particles in the whole electrode generate a large interfacial area to further facilitate electrolyte decomposition and result in capacity fading during cycling. In contrast, the cycled NCM622 cathode in the AFE remains intact without cracking (Fig. 6d-f, Supplementary Fig. 17b and c). The transmission electron microscopy (TEM) images of NCM622 cycled in the BE show a thick CEI (~25 nm) mixed with broken particles (Fig. 6g). In contrast, a much thinner CEI ( < 10 nm) without broken particles is observed in the AFE (Fig. 6h). We therefore conclude that a robust and Al-rich CEI that is formed in the AFE suppresses the cracking of NCM622 particles. We compared the X-ray diffraction (XRD) patterns of the cathodes cycled in the BE and AFE. The peaks corresponding to the (003) interplanar crystal interplanar are shifted to a lower 2‐theta degree in the AFE (Fig. 6i). This means Al^3+^ may enter the lattice of NCM622. The electronegativity of aluminum is smaller than that of cobalt; additionally, the substitution of cobalt by aluminum will increase the charge density of oxygen and electrostatic repulsion between oxygen, resulting in an increase in the interplanar spacing^54^. In addition, the separation of the (006)/(102) and (108)/(110) peaks (Fig. 6j and k) also comes from the influence of Al^3+^ on the crystal structure of NCM622^55^. The XPS (Fig. 6l) and energy dispersive spectroscopy (EDS) (Supplementary Fig. 17d-f) results confirm that the Al-O (75.7 eV) composition including Al_2_O_3_ is evenly distributed on the surface of the NCM622 particles. The total Al content remains at 4.0% of all elements and does not change much with the etching depth (Supplementary Fig. 18), further indicating uniform distribution of Al-O in the CEI film. The detailed C 1 *s*, O 1 *s* and F 1 *s* XPS spectra of the CEI are shown in Supplementary Fig. 19. TOF-SIMS was also performed to provide more information about the composition of the CEI (Fig. 6m and n, Supplementary Figs. 20 and 21). Similar to the LMA, the aluminum-containing species are in close contact with NCM622. More aluminum-containing species and LiF_2_^−^ are formed owing to the uniform coating on the NCM622 particles and the decomposition of electrolyte components at high voltage. It could be speculated that the inhibition of the surface morphology evolution and phase transformation is due to the doped Al^3+^ forming a robust, Al-rich CEI.

**Fig. 7:**
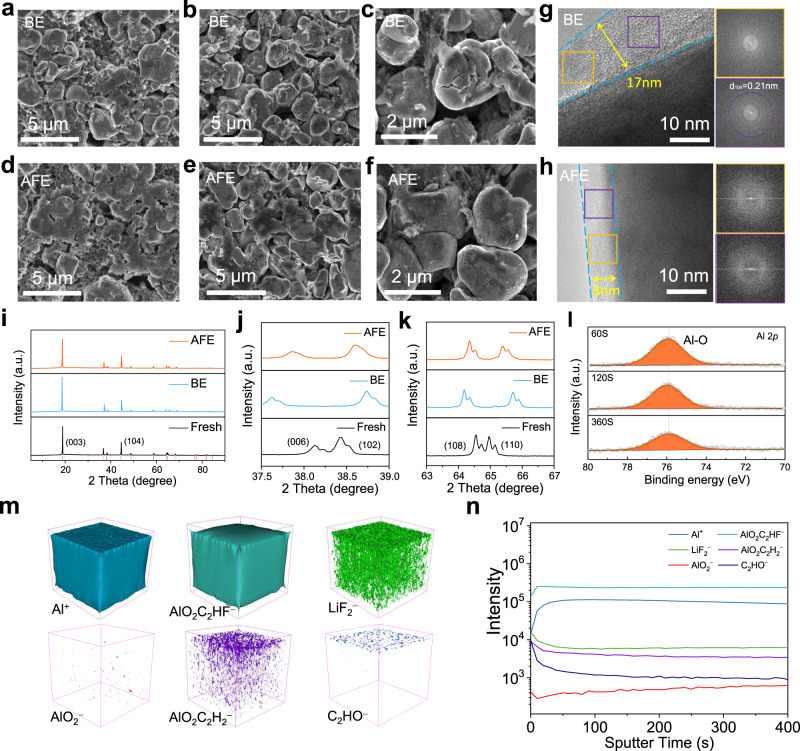
Physicochemical characterization of the cycled high-loading NCM811 cathodes with different electrolytes. SEM images of the NCM811 cathode surface after 50 cycles in the BE (**a**) and AFE (**d**). SEM images of the NCM811 cathodes cross-sectioned after 50 cycles in the BE (**b** and **c**) and AFE (**e** and **f**). TEM analyses of the NCM811 particles after 50 cycles in the BE (**g**) and AFE (**h**). XRD of the NCM811 cathodes (**i**–**k**) before and after 50 cycles in the BE and AFE. Al 2 *p* (**l**) XPS spectra of the NCM811 cathodes in the AFE. TOF-SIMS 3D reconstruction of the sputtered volume (**m**) and the depth profiling of several secondary ion fragments (**n**) of Al^+^, AlO_2_C_2_HF^−^, LiF_2_^−^, AlO_2_^−^, AlO_2_C_2_H_2_^−^ and C_2_HO^−^ on the NCM811 surface after 50 cycles. All the cells were charged and discharged between 3.0 and 4.5 V at 60 mA g^−^^1^ for 50 cycles, and the voltage of the cell was 3.0 V before disassembly.

The cycled NCM811 cathodes (charged and discharged between 3.0 and 4.5 V at 60 mA g^−^^1^ for 50 cycles, and the voltage of the cell was 3.0 V before disassembly) with high mass loading in different electrolytes were also characterized via ex-situ postmortem electrode measurements and analyses to clarify the cycling stability mechanisms of the Li | |NCM811 full batteries (Fig. 7). Cracks can be observed in the surface and cross-section of the cathode cycled with the BE (Fig. 7a-c). The surfaces of the cathodes with AFE show a protective layer, which can effectively inhibit side reactions at the interphase and reduce the formation of internal cracks (Fig. 7d). It can be seen from the cross-section SEM micrographs that the NCM811 particles remain intact with no cracks (Fig. 7e and f). The TEM image shows that a thick (~17 nm) and nonuniform CEI containing broken particles are formed in the BE (Fig. 7g). In contrast, the CEI in the AFE has a thinner and more uniform thickness (~8 nm) and does not contain any NCM fragments (Fig. 7h). The structural change of the NCM811 cathode with the BE and AFE after 50 cycles was examined by XRD. As shown in Fig. 7i-k, the (003) peak in the AFE does not shift to a lower 2‐theta degree, indicating that the influence of Al-doping on the NCM811 cathode is less than that of NCM622, which may be related to the different crystal structures of NCM811 and NCM622. Correspondingly, the separation of the (006)/(102) and (108)/(110) peaks in the AFE is closer to that in the fresh cathode, showing improved structural stability compared to that in the BE^56^. Similar to NCM622, XPS confirms that the Al-O (75.7 eV) composition including Al_2_O_3_, is evenly distributed on the surface of the NCM811 particles (Fig. 7l, Supplementary Fig. 22a). The detailed F 1 *s*, O 1 *s*, and C 1 *s* XPS spectra of the CEI are shown in Supplementary Fig. 22b-d. The TOF-SIMS results show that the inner layer of the CEI is rich in aluminum-containing species, which is similar to that of NCM622. (Fig. 7m and n, Supplementary Figs. 23 and 24).

To summarize, we developed a dual-function electrolyte through polymerization reaction between Al(EtO)_3_ and FEC, which facilitates the formation of robust and ionically conductive protective interphases on both the LMA and NCM cathode. On the LMA side, the SEI with its uniform Al metal and Al_2_O_3_ distribution, allow a more uniform transport and deposition of Li^+^, and results in the formation of densely packed Li. On the NCM cathode side, the CEI containing Al_2_O_3_ mitigates the problem of structural deterioration. Therefore, under practical conditions (NCM811 loading is ~4.0 mAh cm^−^^2^, E/C and N/P ratio are 3.4 g Ah^−^^1^ and 2.13, respectively), the Li | |NCM811 cell exhibits a high capacity retention of 80.3% after 130 cycles, and the calculated energy density is approximately 350 Wh kg^−^^1^.

## Methods

### Materials

The commercial Li[Ni_0.6_Co_0.2_Mn_0.2_]O_2_ (NCM622) electrode (cathode loading is 21.6 mg cm^−^^2^ (active material ratio is 96.5 wt.%, the thickness is 70 μm) and the commercial Li[Ni_0.8_Co_0.1_Mn_0.1_]O_2_ (NCM811) electrode (cathode loading is 21.5 mg cm^−^^2^, active material ratio is 94.5 wt.%, the thickness is 70 μm) were provided by Guangdong Canrd New Energy Technology Co., Ltd. The NCM622 cathode with mass loading of 2.4 mg cm^−^^2^ was prepared by following steps. The mixture of 80 wt.% NCM622 powder, 10 wt.% acetylene black and 10 wt.% polyvinylidene difluoride (PVDF) was manually grinded in an agate mortar for 30 mins and then dissolved in N-methyl-2-pyrrolidone (NMP) by stirring for 4 h to form a slurry. The electrode slurry was then pasted on Al foil (99.35%, 16 μm, HF-Kejing Co., Ltd., China) and dried at 80 °C under vacuum for 12 h. The basic electrolyte (BE) (1 M LiPF_6_ in ethylene carbonate (EC) and diethyl carbonate (DEC) (1:1 by volume) (H_2_O < 10 ppm)) and fluoroethylene carbonate (FEC) (H_2_O < 10 ppm) came from Suzhou Qianmin Chemical Reagent Company. All materials were used as received.

### Preparation of the Al(EtO)_3_-containing functional electrolyte (AFE)

The production details of the Al(EtO)_3_ nanowires are as follows^37^. A graphite crucible with aluminum powder (0.3 g) and Li particles (0.085 g) (1:1 atom ratio) was heated at 800 °C for 30 min in a muffle furnace and naturally cooled to obtain a Li-Al alloy plate. The alloy plate was polished and reacted with ethanol at 60 °C for 30 h to obtain an Al(EtO)_3_ nanowire gel. To prepare the AFE, 0.4 wt.% Al(EtO)_3_ nanowires and 5 vol.% FEC were added into BE and then treated with ultrasonication (DTC-15J, the power of the ultrasound was 150 W) at 35 °C for 3 h before use. The Al(EtO)_3_ nanowires were dispersed in the electrolyte solution. The costs of reagents for preparing Al(EtO)_3_ are shown in Supplementary Table 5. For comparison, an FEC-containing electrolyte (FE) was prepared by adding 5 vol.% FEC in to the BE. To explore the reaction mechanism of Al(EtO)_3_ and FEC, two electrolyte solutions with different concentrations were prepared. One sample was made by adding 40 mg of Al(EtO)_3_ to 1 mL of FEC reagent (Al(EtO)_3_/FEC), while the other sample was prepared by adding 40 mg of Al(EtO)_3_ and 152 mg of LiPF_6_ salt (99.9%, Suzhou Qianmin Chemical Reagent Company) into 1 mL of FEC reagent (Al(EtO)_3_/LiPF_6_/FEC). The two solutions were sonicated (DTC-15J, the ultrasound power of 150 W) at 35 °C for 3 h and then allowed to homogenize for five days.

### Fabrication of the cells

The NCM622 cathode (diameter of 12 mm, thickness of 70 μm), matching commercial Li metal anode (99.95%, 400 μm, China Energy Lithium Co., Ltd.), the NCM811 cathode (diameter of 12 mm, thickness of 70 μm) matching the thin Li metal anode (~40 μm, rolled from 400 μm commercial Li metal anode by hand), separator (Celgard 2500 porous polypropylene film) were assembled into CR2032 coin cells in an Ar-filled glove box (Mikrouna) (H_2_O < 0.1 ppm, O_2_ < 0.1 ppm). The amounts of electrolyte injected were 60 and 12 μL for the Li | |NCM622 and Li | |NCM811 coin cells, respectively. The Li | |Li and Li | |Cu (9 μm Cu metal foil with a purity of 99.8% came from Guangdong Canrd New Energy Technology Co., Ltd.) coin cells were assembled using the same method with 30 μL of electrolyte. All the cells were tested with freshly prepared electrolyte solutions.

### Physicochemical measurements

The morphology and composition of the materials were analyzed using scanning electron microscopy (SEM, Auriga-4523, Zeiss, Germany) and transmission electron microscopy (TEM, FEI Tecnai G2 F30) at an acceleration of 300 kV. The surface chemical state of the cathodes was analyzed through X-ray photoelectron spectroscopy (XPS, ESCALab250), and the etching times were 0, 10, 60, 120, 180, 240, 360 and 480 s. Fourier transform infrared (FTIR) spectroscopy was performed with a NICOLET 6700 instrument (Thermo, America) at a resolution of 0.2 cm^−^^1^. Solid-state NMR spectroscopy was performed at 25 °C and spun at 12 kHz on a Bruker AVANCE 400 superconducting Fourier transform nuclear magnetic resonance instrument. Differential scanning calorimetry (DSC) and thermogravimetric analysis (TGA) were performed at a heating rate of 10 °C min^−^^1^ in a N_2_ atmosphere with a PerkinElmer Pyris Diamond TG/DTA thermal analyzer. X-ray diffraction (XRD) was conducted with an Empyrean powder X-ray diffractometer using a Cu Kα radiation source. Atomic force microscopy (AFM) measurements were performed with a Bruker Dimension Fastscan instrument. Time-of-flight secondary ion mass spectrometry (TOF-SIMS) was performed with a PHI nanoTOF II times-of-flight SIMS instrument. Prior to analysis, the cells were disassembled in Ar-filled glove box (Mikrouna) (H_2_O < 0.1 ppm, O_2_ < 0.1 ppm) and the electrode surfaces were rinsed with 1 mL of Diethyl carbonate (DEC, 99.98%, H_2_O < 10 ppm, Suzhou Qianmin Chemical Reagent Company). After drying at 25 °C under vacuum for 3 h, the electrodes were transferred by the transfer sample holder (Thermo Fisher Scientific) with an Ar-filled or the vacuum container (in-house produced) to isolate the air.

### Electrochemical characterizations

To obtain the cycling and rate performance of the fabricated coin cells, they were placed in an incubator to maintain a constant operating temperature of 30±1 °C and measured at with a battery test system (LAND CT-2001A, Land Electronic Co., Ltd., Wuhan). Electrochemical impedance spectroscopy (EIS) was performed at 30 °C with an electrochemical workstation (IVIUM Vertex. One. EIS) (potentiostatic mode ranged from 10 mHz to 100 kHz at an amplitude of 5 mV, 10 data points per decade, and the open-circuit voltage time applied before carrying out the EIS measurements was 30 s).

## Supplementary information

Supplementary information

## Data Availability

The data that support the findings of this study are included in the paper and its Supplementary Information.
