# Supplementary material for: A dual-function liquid electrolyte additive for high-energy non-aqueous lithium metal batteries
Source: Nat Commun. 2022 Mar 11;13:1297. doi: 10.1038/s41467-022-28959-5 (PMC8917126; doi:10.1038/s41467-022-28959-5)
Supplement: Supplementary file 1 — Supplementary information [file 41467_2022_28959_MOESM1_ESM.pdf]

Supplementary Information

A dual-function liquid electrolyte additive for high-energy non-aqueous lithium metal batteries

Yuji Zhang[1], Yuan Wu[1], Huiyi Li[1], Jinghao Chen[1], Danni Lei[1]*, Chengxin Wang[1]*

Address

[1]State Key Laboratory of Optoelectronic Materials and Technologies, School of Materials Science and Engineering, Sun Yat-sen (Zhongshan) University, Guangzhou 510275, China.

E-mail

leidanni@mail.sysu.edu.cn (D. Lei), wchengx@mail.sysu.edu.cn (C. Wang)

Supplementary Figures

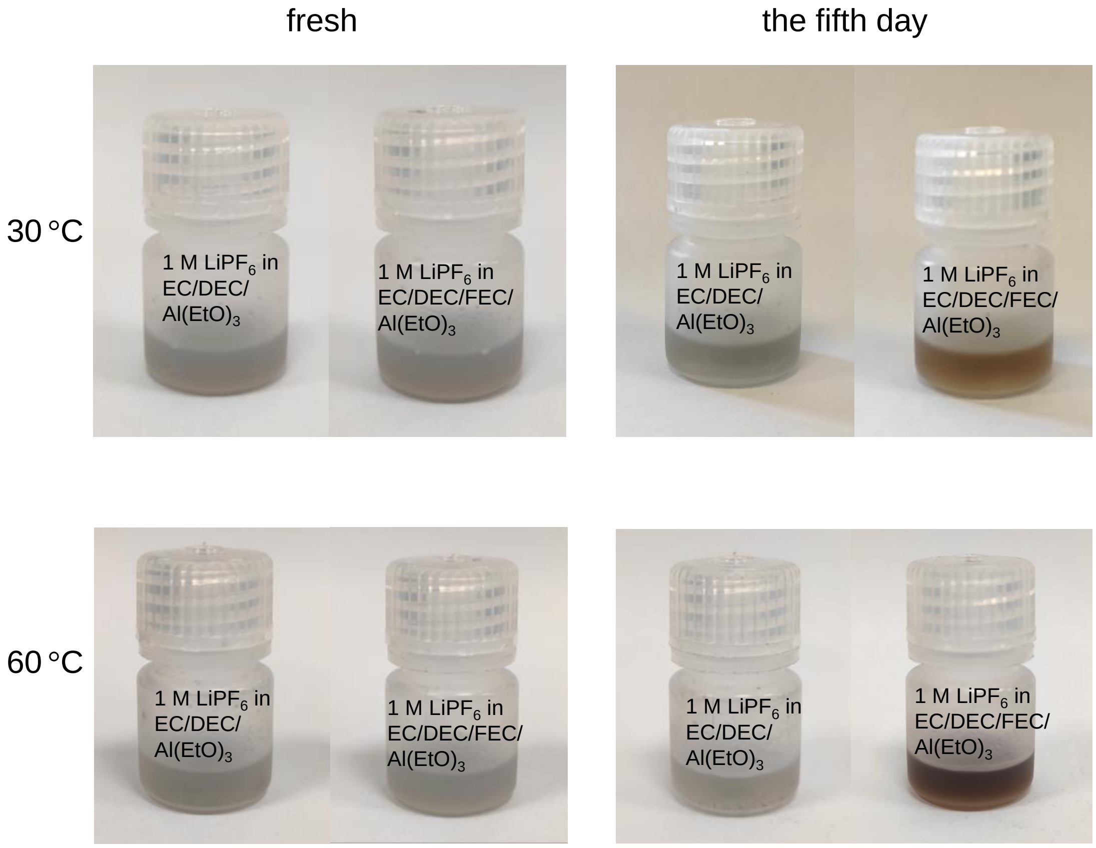

Supplementary Figure 1. Digital photographs showing the colors of different electrolytes before and after standing at 30 and 60 °C for five days. All the electrolyte solutions were freshly prepared and stored in an Ar-filled glovebox.

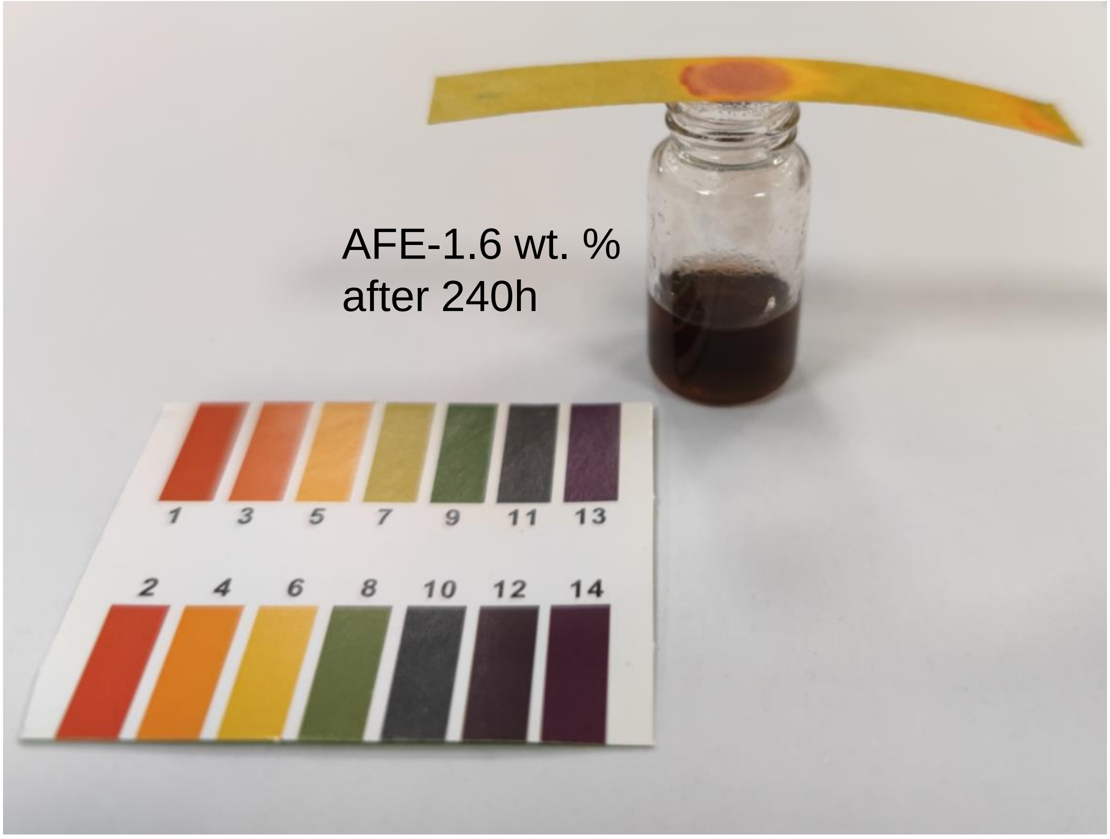

Supplementary Figure 2. The photographs of pH test paper used to test gases produced by AFE-1.6 wt.% electrolyte in air.

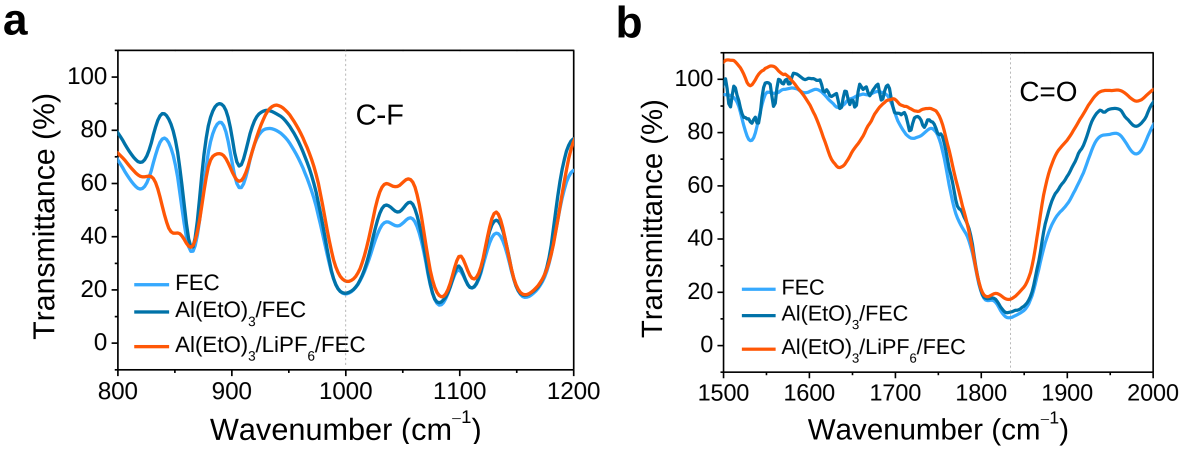

Supplementary Figure 3. FITR spectra of the FEC reagent and supernatant of $Al(EtO)_3$/FEC, $Al(EtO)_3$/$LiPF_6$/FEC solutions.

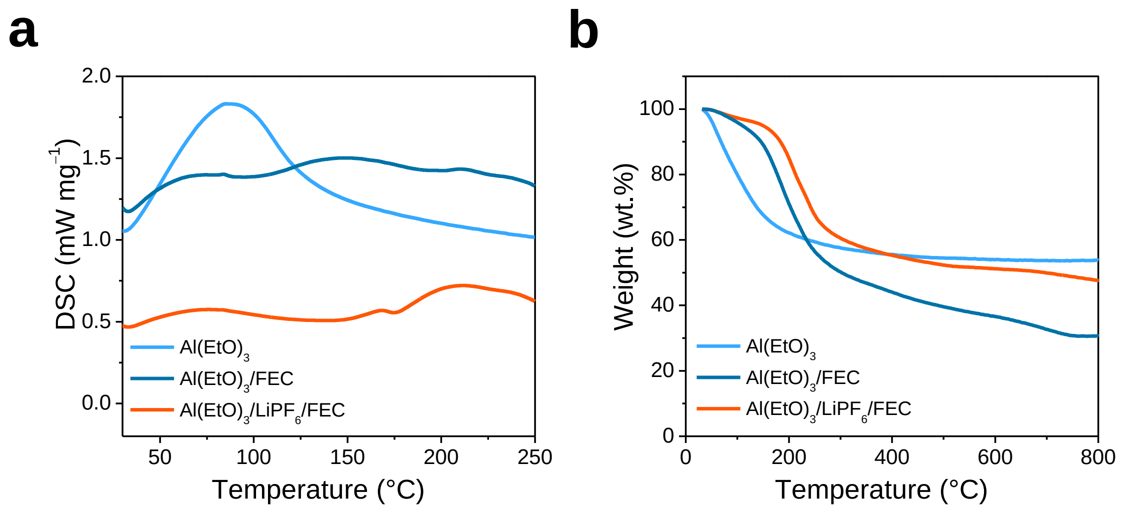

Supplementary Figure 4. Thermal stability analysis of different samples. Differential scanning calorimetry (**a**) and thermogravimetric analysis curves (**b**) of $Al(EtO)_3$ and precipitates obtained from $Al(EtO)_3$/FEC and $Al(EtO)_3/LiPF_6$/FEC solutions at a heating rate of 10 °C min^{-1} in N_2.

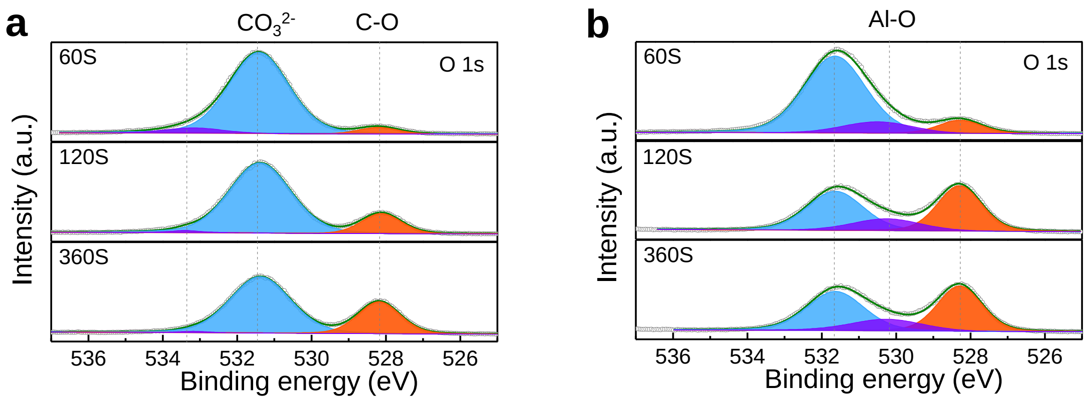

Supplementary Figure 5. XPS analysis of lithium metal anode cycled in different electrolyte systems. XPS O 1*s* spectra of lithium metal anode in the FE (**a**) and the AFE (**b**) after 10 cycles at the current density of 1 mA cm^{-2}.

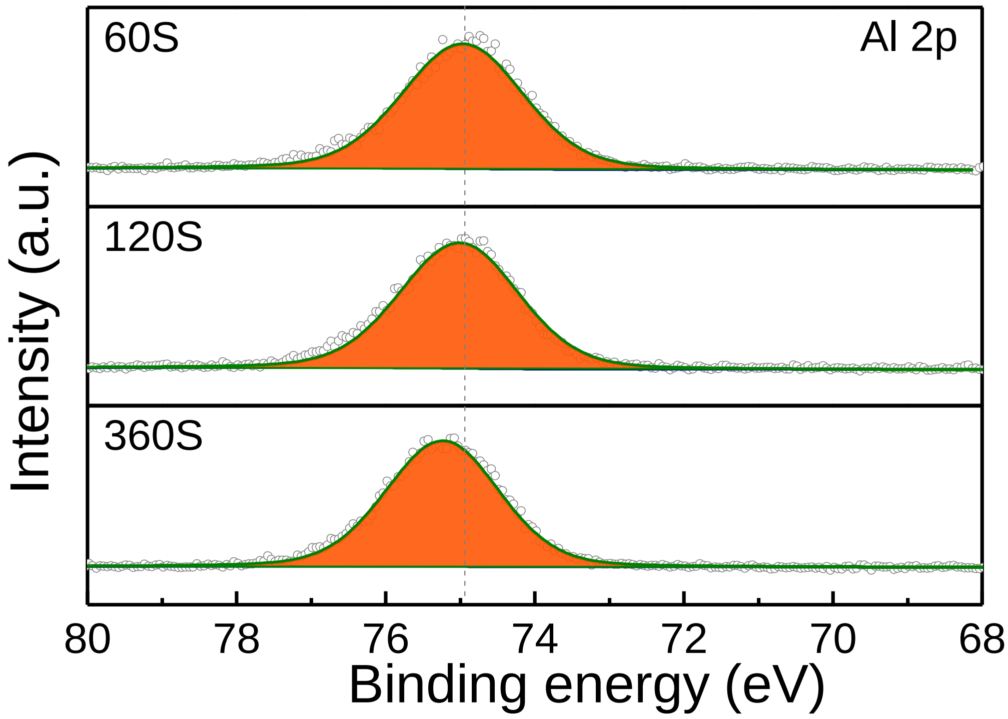

Supplementary Figure 6. Al 2*p* XPS spectra of commercial Al_2O_3 powder.

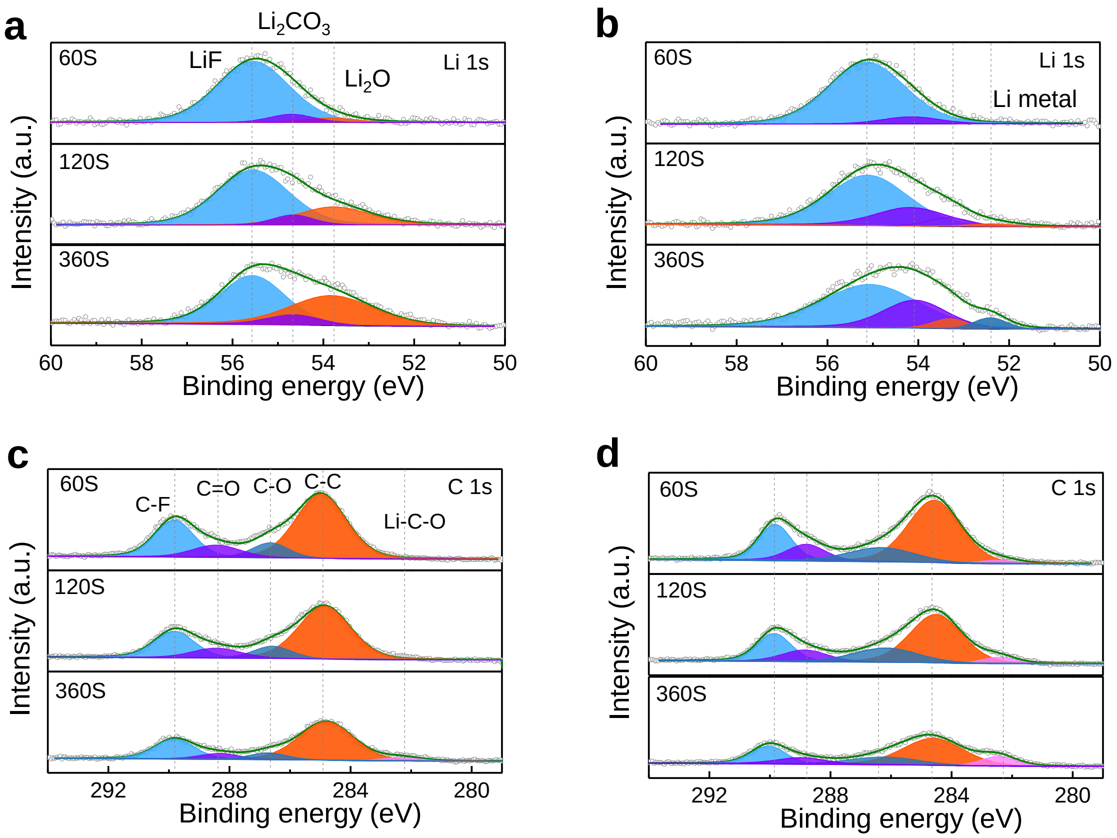

Supplementary Figure 7. XPS analysis of lithium metal anode cycled in different electrolyte systems. XPS Li 1*s* spectra of lithium metal anode in the FE (**a**) and the AFE (**b**), XPS C 1*s* spectra of lithium metal anode in the FE (**c**) and the AFE (**d**) after 10 cycles at 1 mA cm^{-2}.

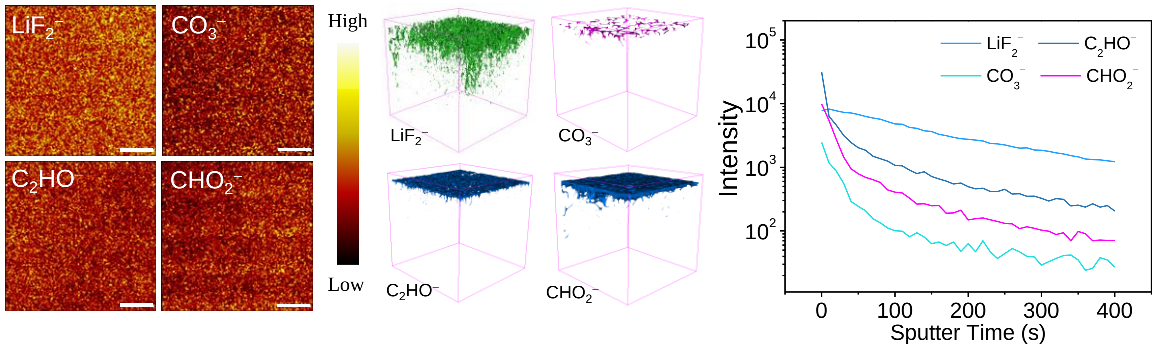

Supplementary Figure 8. TOF-SIMS chemical mapping and 3D reconstruction of the sputtered volume of several secondary ion fragments on Li surface in the AFE. The scale bars are 20 μm.

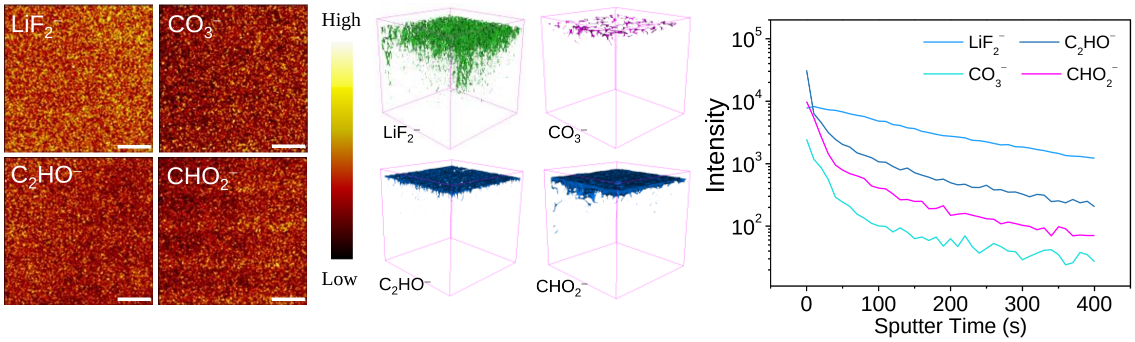

Supplementary Figure 9. TOF-SIMS chemical mapping and 3D reconstruction of the sputtered volume and the depth profiling of several secondary ion fragments on Li surface in the FE. The scale bars are 20 μm.

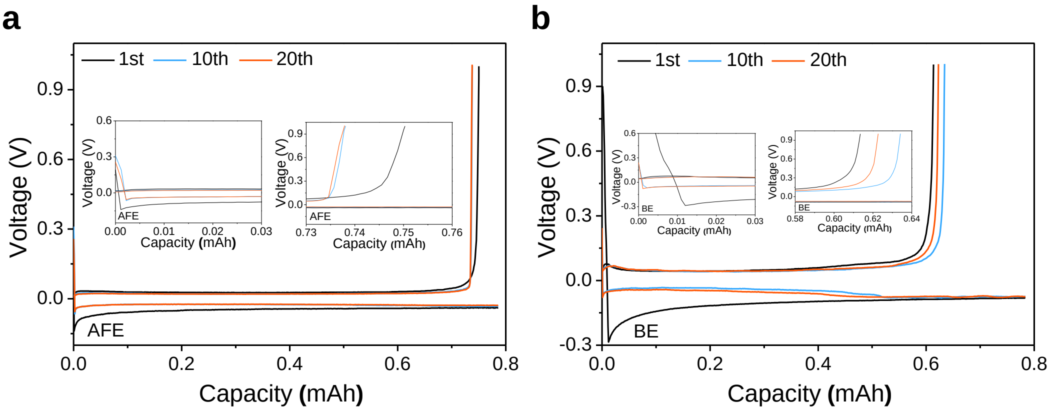

Supplementary Figure 10. Voltage profiles of Li||Cu cells using the AFE (**a**) and the BE (**b**).

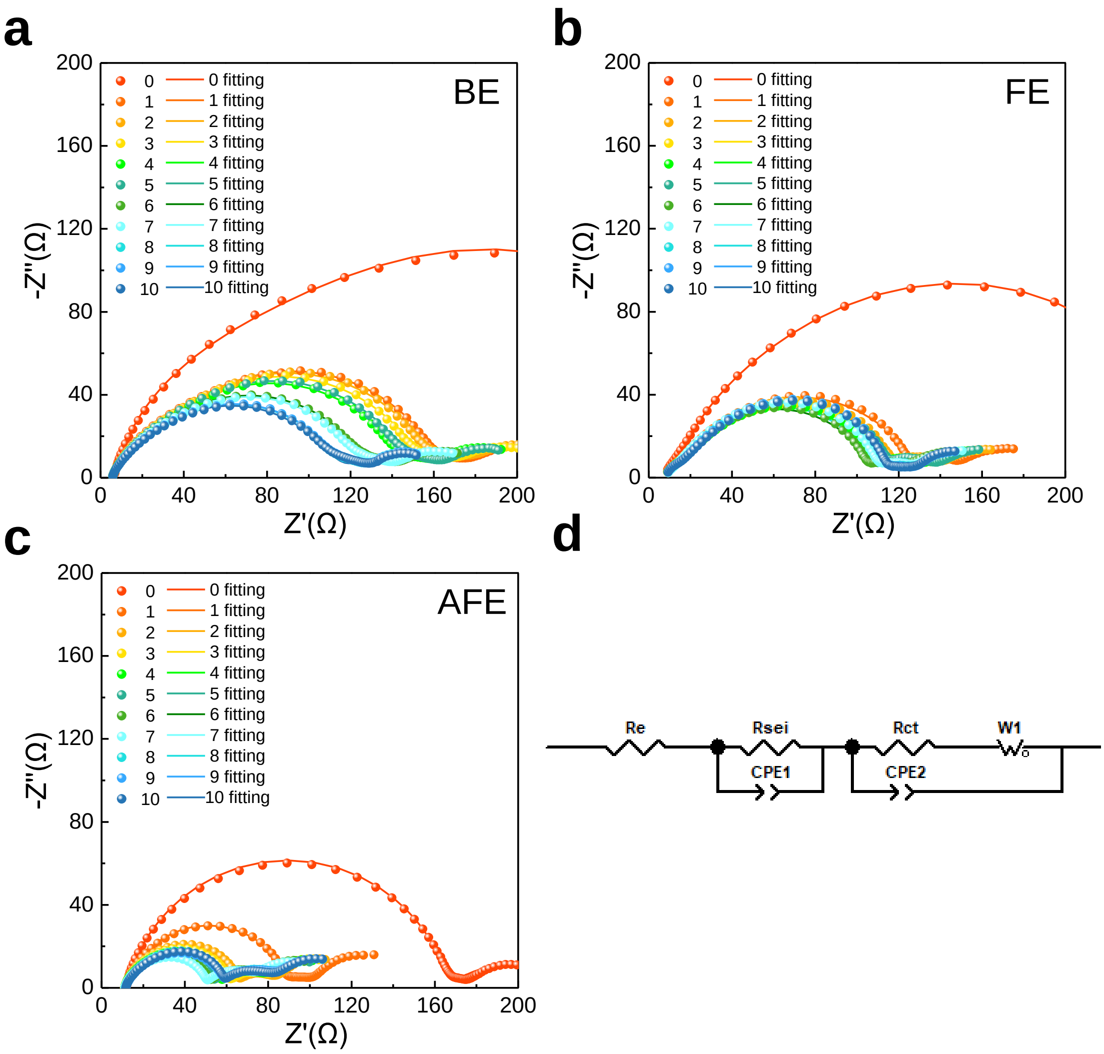

Supplementary Figure 11. Electrochemical impedance spectra of the Li||Li cells using the BE (**a**), FE (**b**) and AFE(**c**) with an increasing cycle number (0-10 cycle) and the equivalent circuit (**d**). The current density is 1.0 mA cm^{-2} and capacity is 1 mAh cm^{-2}.

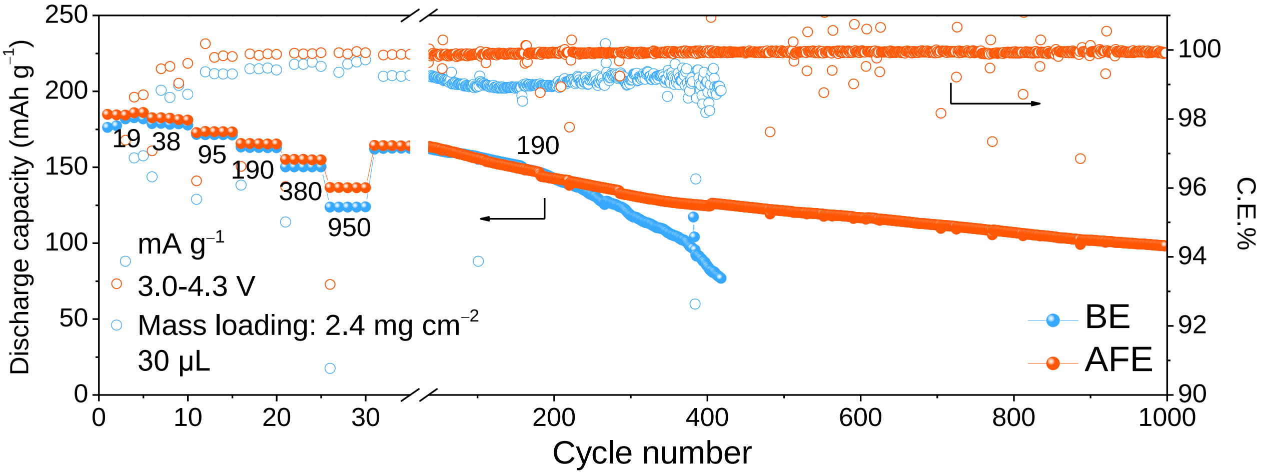

Supplementary Figure 12. Cycling performance of Li||NCM622 cells with different electrolyte at 19 mA g^{-1}, 38 mA g^{-1}, 95 mA g^{-1}, 190 mA g^{-1}, 380 mA g^{-1} and 950 mA g^{-1} for 5 cycles and finally stabilized at 190 mA g^{-1} (same charging and discharging rates were used).

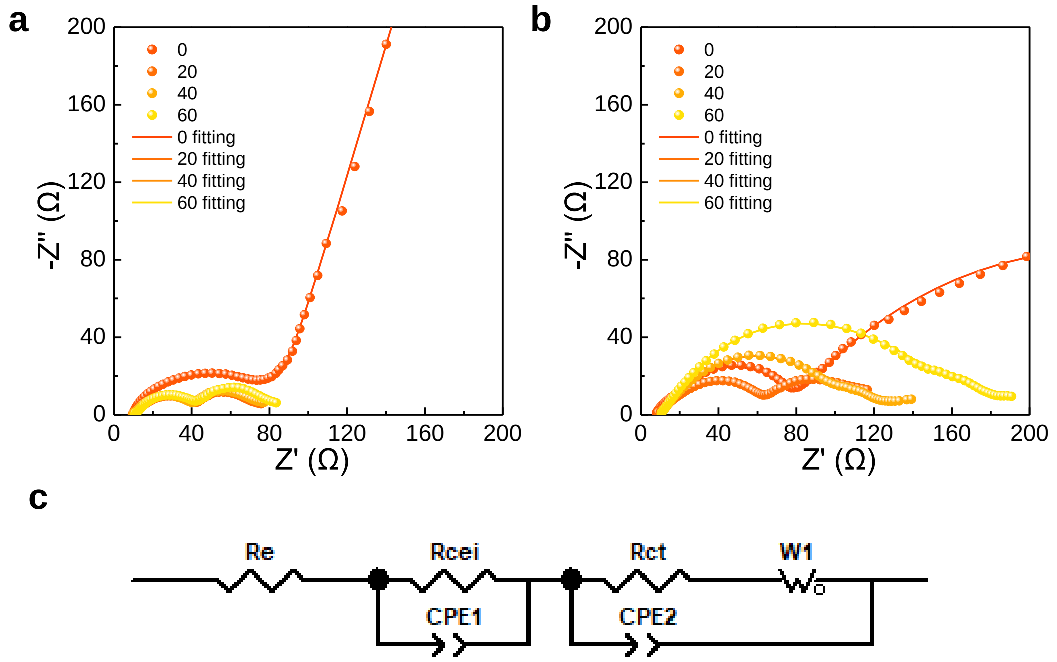

Supplementary Figure 13. Electrochemical impedance spectra of high-loading Li||NCM622 cells (mass loading is 21.6 mg cm^{-2}) with the AFE (**a**) and the BE (**b**) before and after different cycles (open circuit voltage was 3.6 V) and the equivalent circuit (**c**).

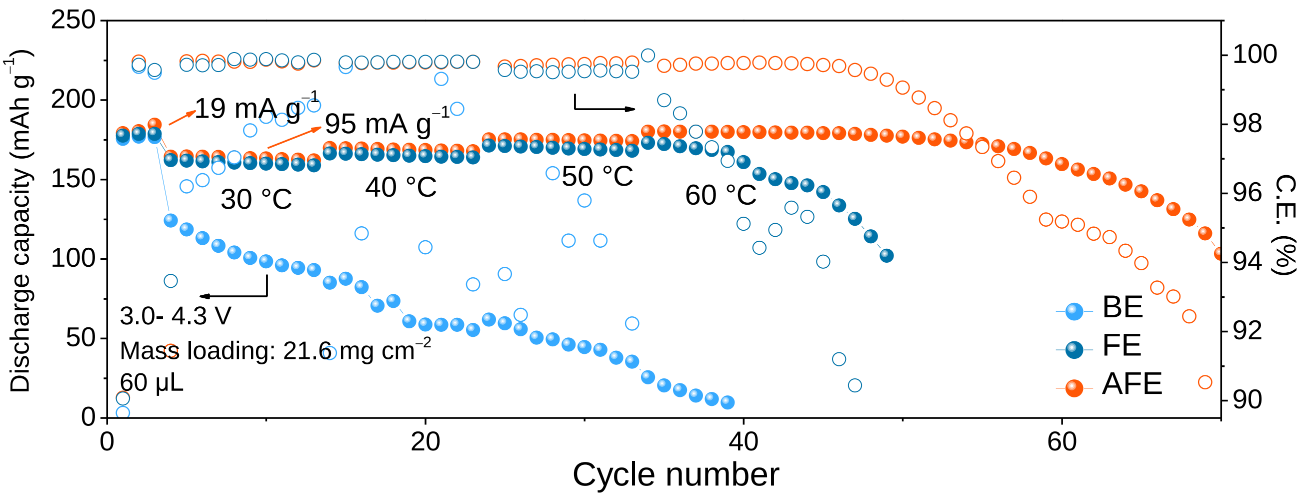

Supplementary Figure 14. Cycling performances of Li||NCM622 cells with different electrolytes from 30 to 60 °C.

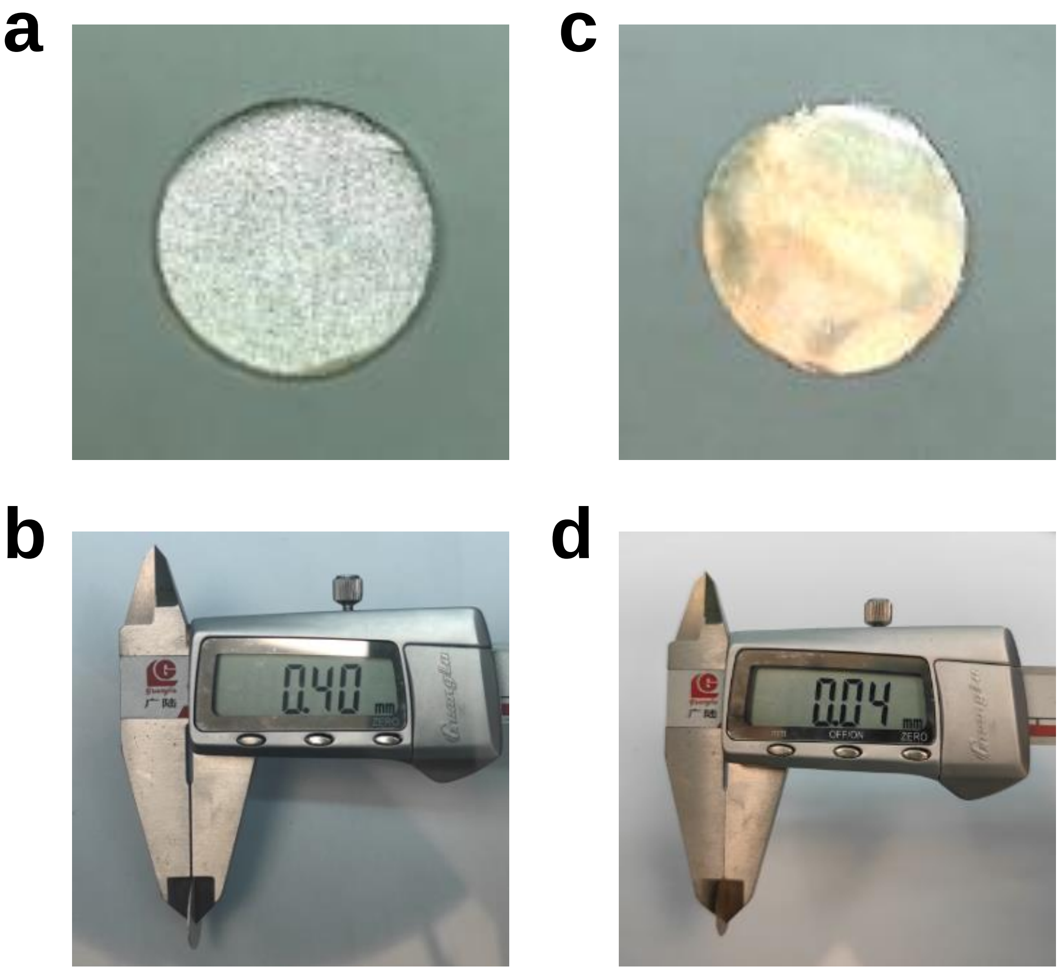

Supplementary Figure 15. The photographs and thicknesses of commercial Li metal anode (**a, b**) and Li metal anode that we used to assemble the Li||NCM811 cells (**c, d**).

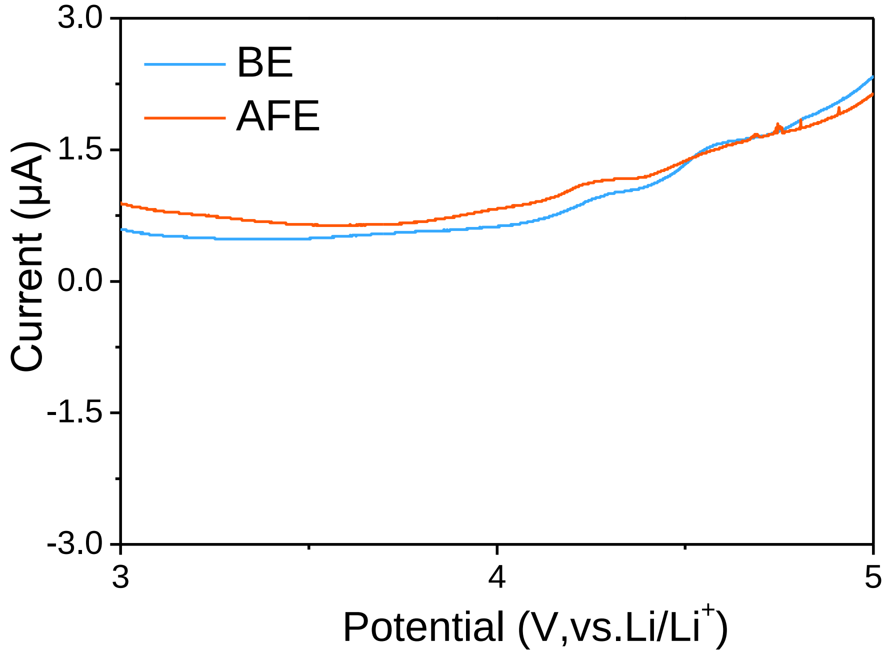

Supplementary Figure 16. Linear sweep voltammograms at 0.2 mV s^{-1} with the BE and AFE with a stainless steel (SS) working electrode.

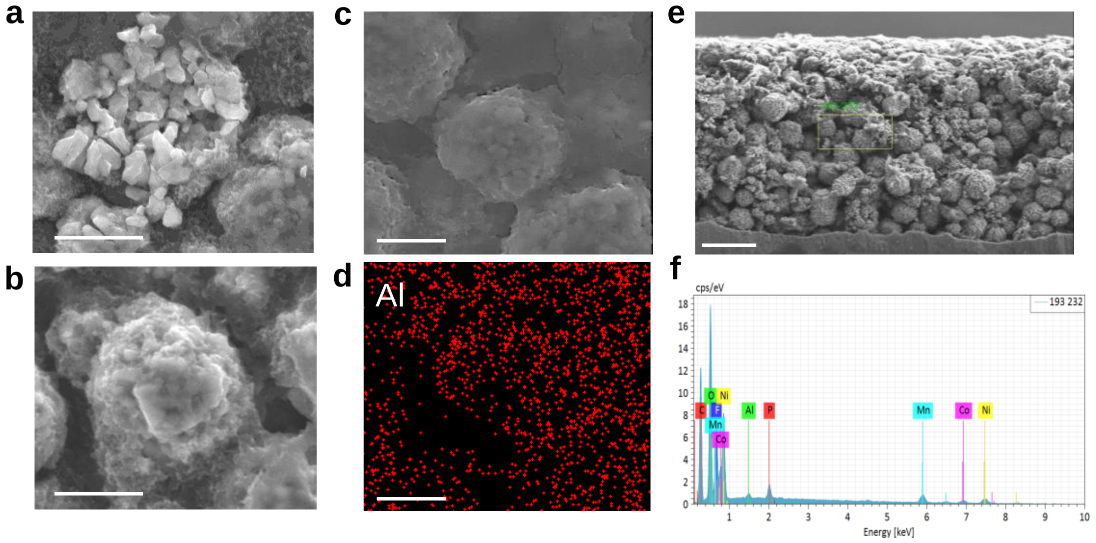

Supplementary Figure 17. Morphology and elemental analysis of NCM622 cathode in different electrolytes system. SEM images of NCM622 cathodes surface in the BE (**a**) and AFE (**b** and **c**). Element mapping images of NCM622 cathode (**d**). Cross-section SEM image of NCM622 cathode in the AFE (**e**). Element content of the area selected by a box in Figure 16e (**f**). All the cells were cycling for 100 times at the specific current of 95 mA g^{-1}. (Scale bars: **a-d** 5 μm; **e** 20 μm)

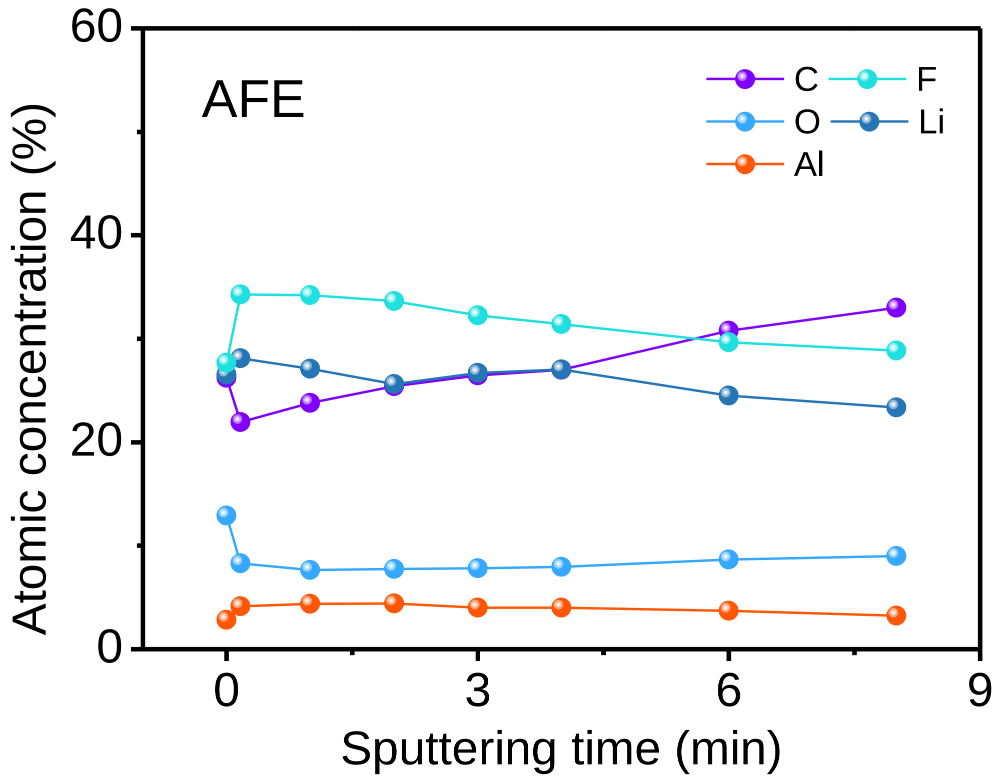

Supplementary Figure 18. Atomic ratio of elements in CEI on NCM622 cathode at different sputtering times.

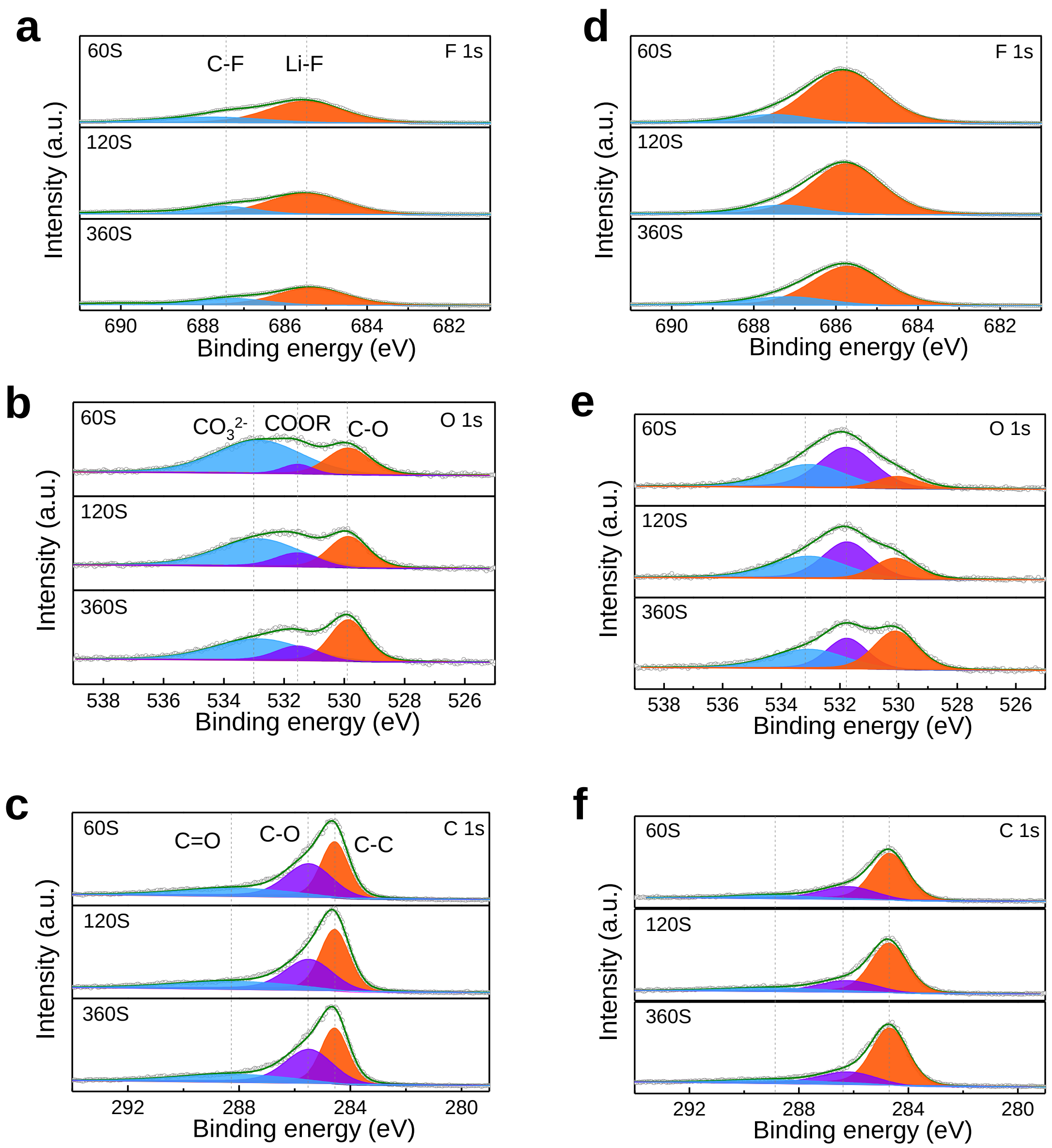

Supplementary Figure 19. XPS analysis of NCM622 cathode cycled in different electrolyte systems. F 1*s*, O 1*s* and C 1*s* spectra of NCM622 cathode in BE (**a-c**) and AFE (**d-f**) after cycling for 100 times at the specific current of 95 mA g^{-1}.

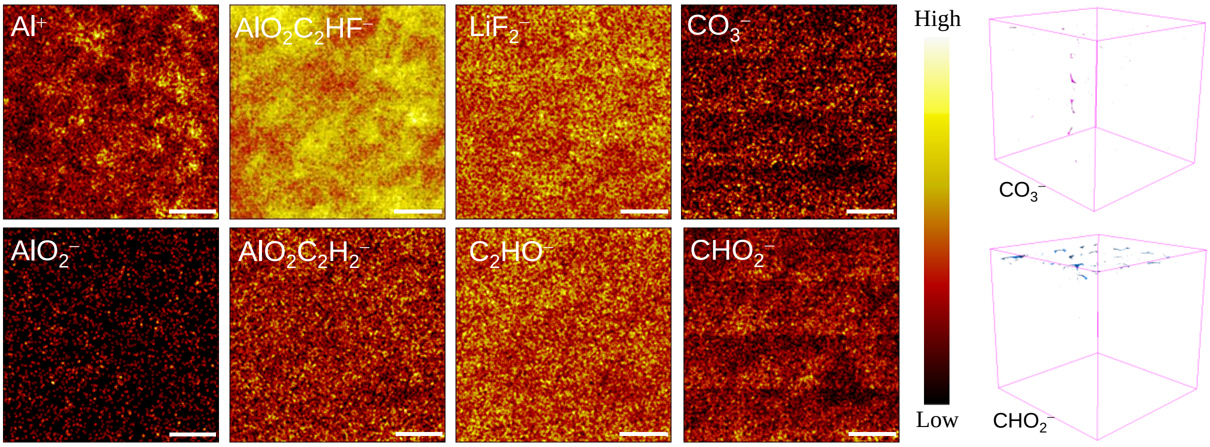

Supplementary Figure 20. TOF-SIMS chemical mapping and 3D reconstruction of the sputtered volume of several secondary ion fragments on NCM622 surface in the AFE. The scale bars are 20 µm.

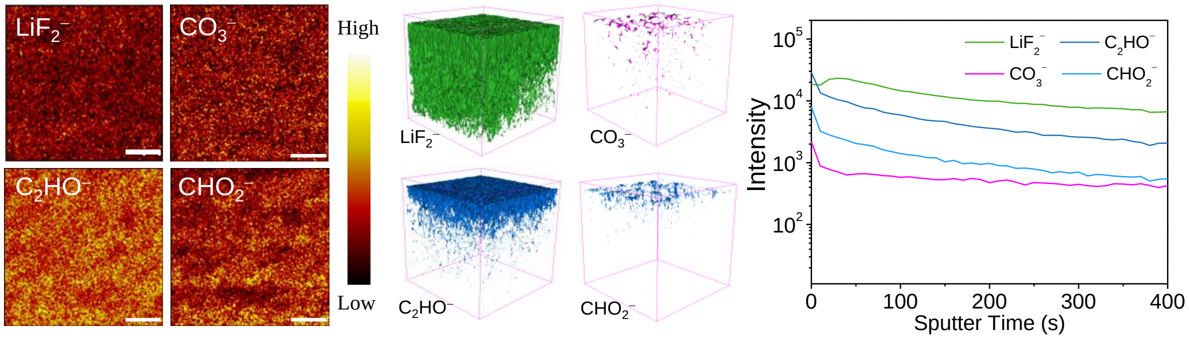

Supplementary Figure 21. TOF-SIMS chemical mapping, 3D reconstruction of the sputtered volume and the depth profiling of several secondary ion fragments on NCM622 surface in the BE. The scale bars are 20 μm.

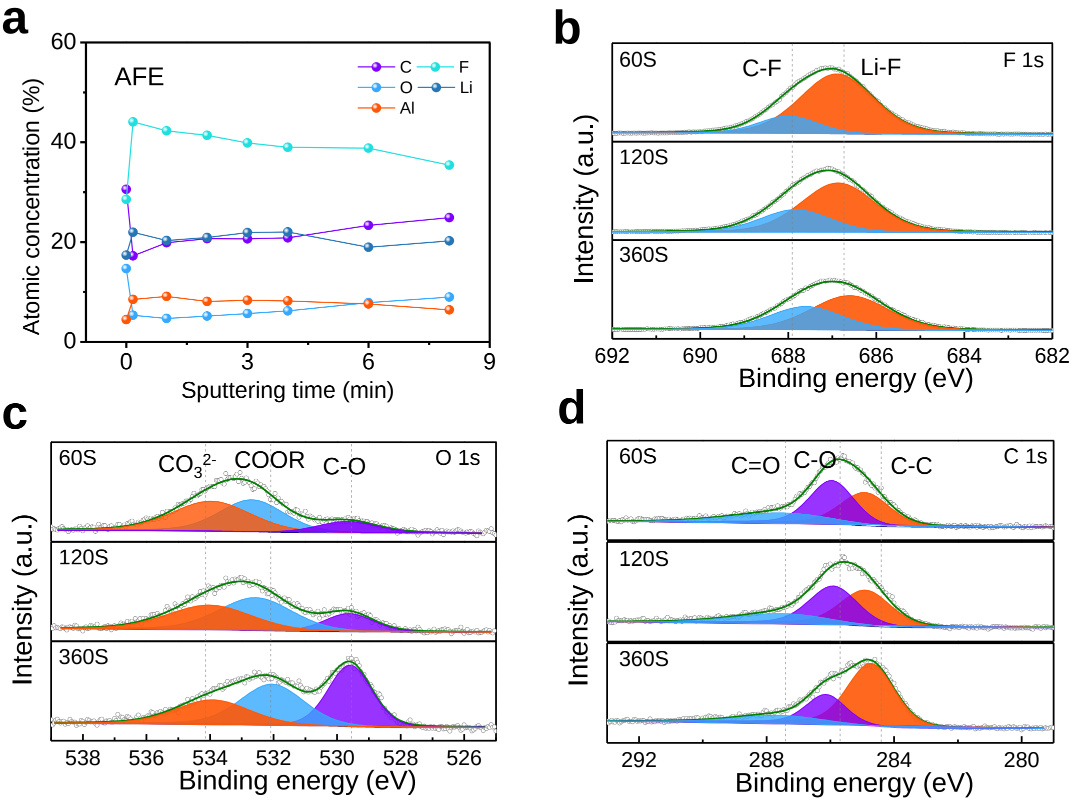

Supplementary Figure 22. Atomic ratio of elements in CEI on NCM811cathode at different sputtering times (**a**). XPS F 1*s* spectra of NCM811 cathode in the AFE (**b**), XPS O 1*s* spectra of NCM811 cathode in AFE (**c**), XPS C 1*s* spectra of NCM811 cathode in AFE (**d**) after 50 cycles at 60 mA g^{-1}.

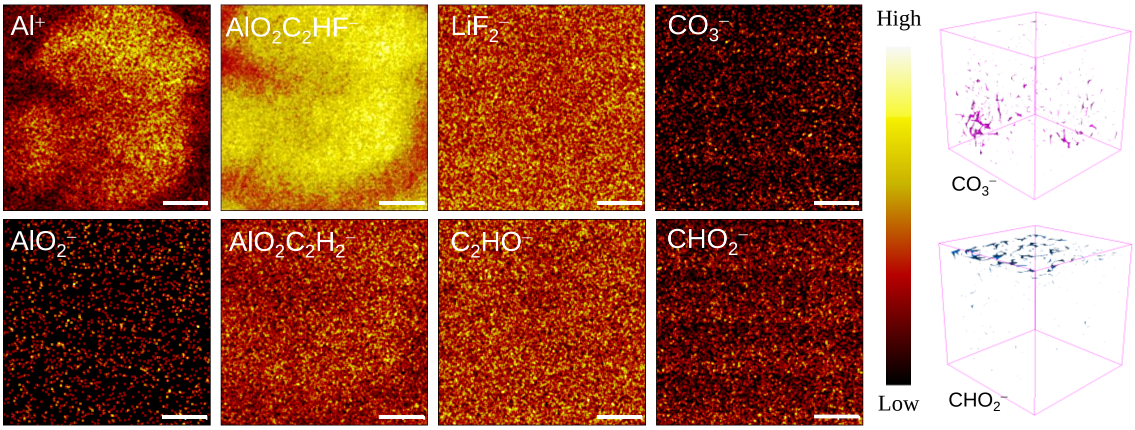

Supplementary Figure 23. TOF-SIMS chemical mapping and 3D reconstruction of the sputtered volume of several secondary ion fragments on NCM811 surface in AFE. The scale bars are 20 μm.

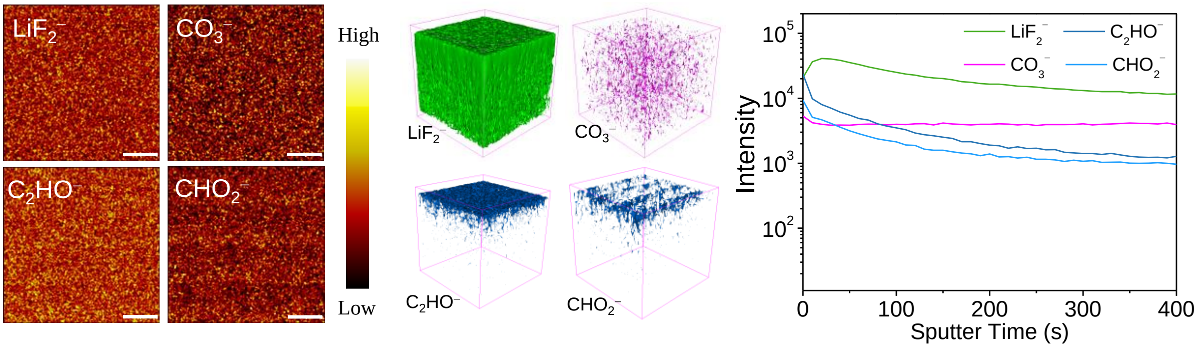

Supplementary Figure 24. TOF-SIMS chemical mapping, 3D reconstruction of the sputtered volume and the depth profiling of several secondary ion fragments on NCM811 surface in the BE. The scale bars are 20 μm.

Supplementary Tables

Supplementary Table 1 Fitted EIS results of Li||NCM622 (mass loading is 21.6 mg cm^{-2}) cells after different cycles.

Sample	R_{CEI} (Ω)	Error (%)
AFE-20	36.2	1.40
AFE-40	31.5	0.97
AFE-60	33.9	1.11
BE-20	53.9	1.24
BE-40	61.6	4.39
BE-60	101.8	2.63

Supplementary Table 2 Cell parameters of NCM811||Li coin cell for 356.23 Wh kg^{-1} according to our measurements.

Parameter		Assigned Value
Cathode areal capacity		3.96 mAh cm^{-2}
Anode areal capacity		8.42 mAh cm^{-2}
Active material ratio		94.5 wt.%
N/P ratio		2.13
E/C ratio		3.4 g Ah^{-1}
Discharge capacity		195.1 mAh g^{-1}
Average discharge voltage		3.78 V
Assigned density	NCM811 cathode	21.5 mg cm^{-2}
	Lithium metal anode	2.18 mg cm^{-2}
	Al foil	3.85 mg cm^{-2}
	AFE	13.46 mg cm^{-2}
	Separator	1.03 mg cm^{-2}
	Total	42.02 mg cm^{-2}
Specific energy		356.23 Wh kg^{-1}

- Except for the specific density, all other values were actually measured.
- The mathematical methods applied to calculate the specific energy values:

$$Specific\ energy = \frac{Cathode\ areal\ capacity * Average\ discharge\ voltage}{Total\ assigned\ density}$$

Supplementary Table 3 Cell parameters of the Li||NCM811 18650 cell for 346.1 Wh kg^{-1}.

Parameter	Assigned Value
Pa- Percentage of active material	94.5 wt.%
Pb- Percentage of binder	3.0 wt.%
Pc- Percentage of conductive carbon	2.5 wt.%
ρa- Density of active material	4.70 g cm^{-3}
ρb- Density of binder	1.80 g cm^{-3}
ρc- Density of conductive carbon	2.26 g cm^{-3}
Ka- Porosity of anode	33%
Kc- Porosity of cathode	30%
ρLi- Density of lithium metal	0.534 g cm^{-3}
ρElec- Density of electrolyte	1.27 g cm^{-3}
Ec- Average cathode potential Vs Li [V]	3.78 V
Ea- Average anode potential Vs Li [V]	0 V
Hc- Thickness of cathode	70 μm
Ha- Thickness of anode	41 μm
D- Specific capacity	195.1 mAh g^{-1}
ρCu- Areal density of Cu Current collector	0.00358 g cm^{-2}
ρAl- Areal density of Al Current collector	0.00270 g cm^{-2}
ρ(Sep+Elec)- Areal density of Separator+Electrolyte	0.00261 g cm^{-2}
Specific energy	413 Wh kg^{-1}
Percentage of packing materials	16.2 wt.%
Specific energy (18650)	346.1 Wh kg^{-1}

• The values of ρa, ρb, ρc, Ka, Kc, ρLi, ρCu, ρAl and Percentage of packing materials were from reported literatures[1, 2] and the values of Pa, Pb, Pc, ρElec, Ec, Ea, Hc, Ha, D and ρ(Sep+Elec) were actually measured.

• The calculation method of energy density of Specific energy is as follows[1]:

$$Mc = \frac{1}{\left(\frac{Pa}{\rho a}\right) + \left(\frac{Pb}{\rho b}\right) + \left(\frac{Pc}{\rho c}\right)}$$

$$Fc = (1 - Kc) * Mc + Kc * \rho Elec$$

$$Fa = (1 - Ka) * \rho Li + Ka * \rho Elec$$

$$Fam = (1 - Kc) * Mc * Pa$$

$$Specific\ energy = \frac{(Ec - Ea) * Hc * D * Fam}{(Hc * Fc) + (Ha * Fa) + \rho Cu + \rho Al + \rho(Sep + Elec)}$$

- The calculation method of energy density of Specific energy (18650) is as follows:

$$Specific\ energy\ (18650) = Specific\ energy * (1 - 16.2\ wt.\%)$$

Supplementary Table 4 Comparison of our work with recent electrolyte works on Li (or Cu)||NCM batteries.

**Electrolyte**	**Based on commercial electrolyte**	**Cathode, mass loading (mg cm^{-2}), active material ratio (wt.%)**	**Cut-off voltage (V)**	**Cathode loading (mAh cm^{-2})**	**Capacity retention rate (cycle number)**	**E/C ratio (g Ah^{-1})**	**N/P ratio**	**Anode and its thickness (μm)**	**Specific energy (Wh kg^{-1})**	**Ref.**
1M $LiPF_6$ in EC/DEC+5%FEC+$Al(EtO)_3$	YES	NCM811, 21.5, 94.5	4.5	3.96	80.3% (130 cycles)	3.4	2.13	Li (41)	356.23	This work
1M $LiPF_6$/EC-DEC-5 wt.% $LiNO_3$-0.4 wt.% $Sn(OTf)_2$	YES	NCM811, 16.7, N/A	4.3	3.34	89.6% (130 cycles)	14	2.64	Li (45)	N/A	3
0.6 M LiTFSI, 0.4 M LiBOB, 50 mM $LiPF_6$, in 4:6 (w/w) EC: EMC	NO	NCM442 10.8, N/A	4.5	1.75	N/A (168 cycles)	54	13.7	Li (120)	N/A	4
7 M LiFSI in FEC	NO	$LiNi_{0.5}Mn_{1.5}O_4$ 14.7, 84	5.0	1.83	78% (130 cycles)	N/A	1.4	N/A	N/A	5
1.2 M LiFSI/TEP-BTFE	NO	NCM622 21.4, 96	4.3	3.79	86% (200 cycles)	3	2.6	Li (50)	300	6
LiFSI-1.2DME-3TTE	NO	NCM811 N/A, N/A	4.5	4.2	80% (155 cycles)	3	2.2	Li (50)	325	7
1 M $LiPF_6$/FEC-EMC-$LiNO_3$-TPFPB	NO	NCM811 17.72, 95	4.5	4.03	80% (140 cycles)	3.4	2.28	Li (46)	295.1	8
1 M LiFSI/FDMB	NO	NCM811 N/A, N/A	4.4	3.9	80% (70 cycles)	2	0	Cu (N/A)	325	9
1 M LiFSI in DMTMSA	NO	NCM811 18.4, 94	4.7	4.67	88.1% (100 cycles)	2.62	0.39	Li (60)	N/A	10

Supplementary Table 5 Costs of reagents.

Role	Grade	Price	Vendor
Lithium metal	≥99.9%	$938/kg	Aladdin
Aluminum metal	≥99.95%	$200/kg	Aladdin
Ethanol	≥99.98%	$13.4/L	Macklin
Aluminum ethoxide (44% yield)	–	$1.16/g	in-house produced

• The cost calculation method of Aluminum ethoxide: [The price of (0.425 g Al+1.5 g Li+100 mL Ethanol)]×44%

• $ is US dollar

Supplementary References

1. Berg, E. J., Villevieille, C., Streich, D., Trabesinger, S.&Novák, P. Rechargeable batteries: Grasping for the limits of chemistry. J. Electrochem. Soc. **162,** A2468-A2475 (2015).
2. Betz, J. et al. Theoretical versus practical energy: A plea for more transparency in the energy calculation of different rechargeable battery systems. Adv. Energy Mater. **9,** 1803170 (2018).
3. Zhang W, Wu Q, Huang J, Fan L, Shen Z, He Y, et al. Colossal granular lithium deposits enabled by the grain-coarsening effect for high-efficiency lithium metal full batteries. *Adv. Mater.* **32,** e2001740 (2020).
4. Zheng J, Engelhard MH, Mei D, Jiao S, Polzin BJ, Zhang J-G, et al. Electrolyte additive enabled fast charging and stable cycling lithium metal batteries. *Nat. Energy* **2,** 17012 (2017).
5. Suo L, Xue W, Gobet M, Greenbaum S, Wang C, Chen Y, et al. Fluorine-donating electrolytes enable highly reversible 5 V-class Li metal batteries. *Proc. Natl. Acad. Sci. U.S.A.* **115,** 1156-1161 (2018).
6. Niu C, Lee H, Chen S, Li Q, Du J, Xu W, et al. High-energy lithium metal pouch cells with limited anode swelling and long stable cycles. *Nat. Energy* **4,** 551-559 (2019).
7. Ren X, Zou L, Cao X, Engelhard MH, Liu W, Burton SD, et al. Enabling high-voltage lithium-metal batteries under practical conditions. *Joule* **3,** 1662-1676 (2019).
8. Li S, Zhang W, Wu Q, Fan L, Wang X, Wang X, et al. Synergistic dual-additive electrolyte enables practical lithium-metal batteries. *Angew. Chem. Int. Ed.* **59,** 14935-14941 (2020).

9. Yu Z, Wang H, Kong X, Huang W, Tsao Y, Mackanic DG, et al. Molecular design for electrolyte solvents enabling energy-dense and long-cycling lithium metal batteries. *Nat. Energy* **5,** 526-533 (2020).

10. Xue W, Huang M, Li Y, Zhu YG, Gao R, Xiao X, et al. Ultra-high-voltage Ni-rich layered cathodes in practical Li metal batteries enabled by a sulfonamide-based electrolyte. *Nat. Energy* **6,** 495-505 (2021).
